# Transcriptional regulation of cellobiose utilization by PRD-domain containing Sigma54-dependent transcriptional activator (CelR) and catabolite control protein A (CcpA) in *Bacillus thuringiensis*

**DOI:** 10.3389/fmicb.2024.1160472

**Published:** 2024-01-31

**Authors:** Liangwei Zhang, Hong Xu, Haijian Cheng, Fuping Song, Jie Zhang, Qi Peng

**Affiliations:** State Key Laboratory for Biology of Plant Diseases and Insect Pests, Institute of Plant Protection, Chinese Academy of Agricultural Sciences, Beijing, China

**Keywords:** *Bacillus thuringiensis*, cellobiose, regulation, CelR, CcpA

## Abstract

Cellobiose, a β-1,4-linked glucose dimer, is a major cellodextrin resulting from the enzymatic hydrolysis of cellulose. It is a major source of carbon for soil bacteria. In bacteria, the phosphoenolpyruvate (PEP): carbohydrate phosphotransferase system (PTS), encoded by the *cel* operon, is responsible for the transport and utilization of cellobiose. In this study, we analyzed the transcription and regulation of the *cel* operon in *Bacillus thuringiensis* (*Bt*). The *cel* operon is composed of five genes forming one transcription unit. β-Galactosidase assays revealed that *cel* operon transcription is induced by cellobiose, controlled by Sigma54, and positively regulated by CelR. The HTH-AAA^+^ domain of CelR recognized and specifically bound to three possible binding sites in the *celA* promoter region. CelR contains two PTS regulation domains (PRD1 and PRD2), which are separated by two PTS-like domains-the mannose transporter enzyme IIA component domain (EIIA^Man^) and the galactitol transporter enzyme IIB component domain (EIIB^Gat^). Mutations of His-546 on the EIIA^Man^ domain and Cys-682 on the EIIB^Gat^ domain resulted in decreased transcription of the *cel* operon, and mutations of His-839 on PRD2 increased transcription of the *cel* operon. Glucose repressed the transcription of the *cel* operon and catabolite control protein A (CcpA) positively regulated this process by binding the *cel* promoter. In the *celABCDE* and *celR* mutants, PTS activities were decreased, and cellobiose utilization was abolished, suggesting that the *cel* operon is essential for cellobiose utilization. *Bt* has been widely used as a biological pesticide. The metabolic properties of *Bt* are critical for fermentation. Nutrient utilization is also essential for the environmental adaptation of *Bt*. Glucose is the preferred energy source for many bacteria, and the presence of the phosphotransferase system allows bacteria to utilize other sugars in addition to glucose. Cellobiose utilization pathways have been of particular interest owing to their potential for developing alternative energy sources for bacteria. The data presented in this study improve our understanding of the transcription patterns of *cel* gene clusters. This will further help us to better understand how cellobiose is utilized for bacterial growth.

## 1 Introduction

**Figure 1:**
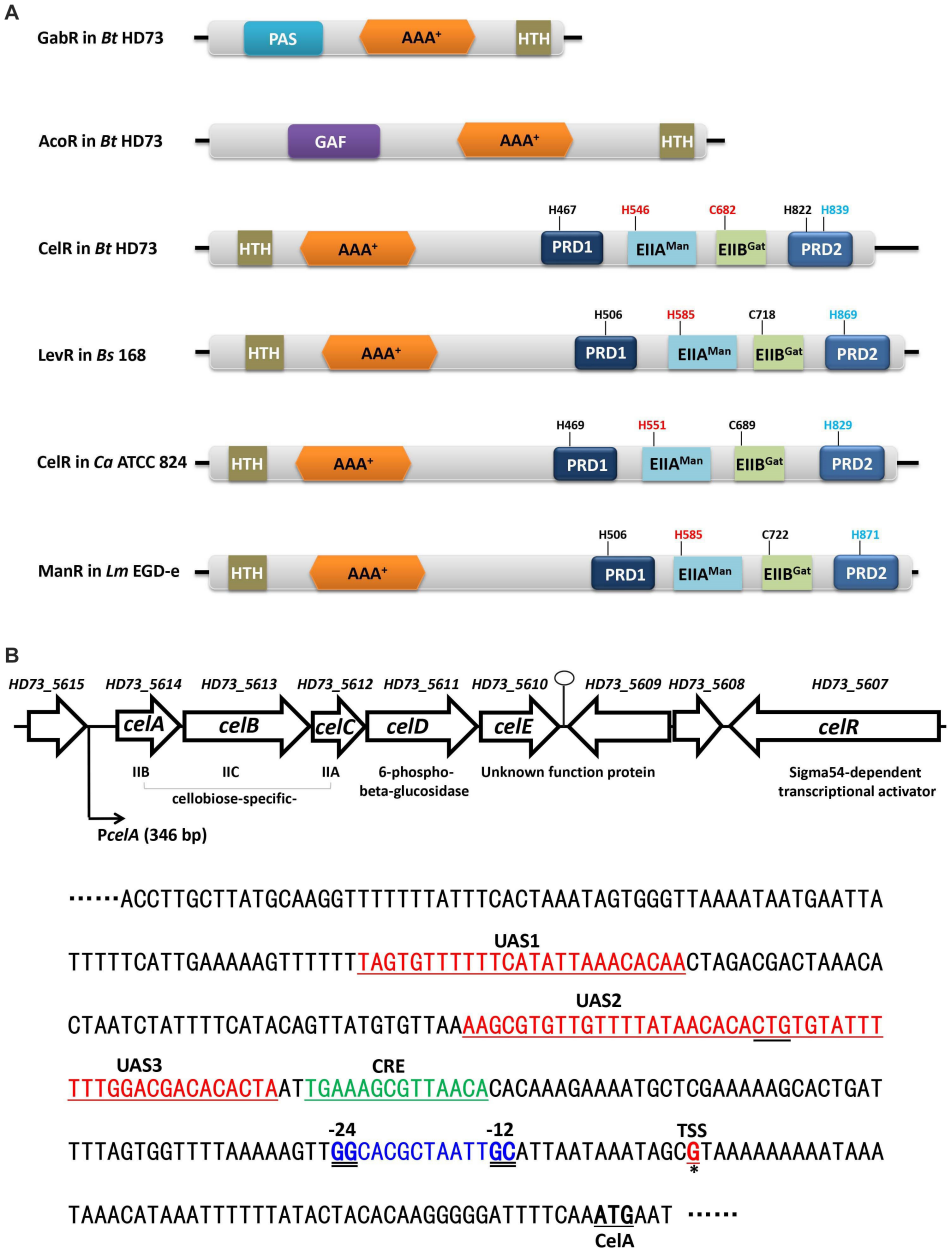
The *cel* gene cluster on the *Bt* HD73 chromosome. **(A)** Schematic representation of GabR and AcoR in *Bt* HD73, and CelR in *Bt* HD73, LevR in *B. subtilis* 168, CelR in *C. acetobutylicum* ATCC 824, and ManR in *L. monocytogenes* EGD-e. The numbers indicate the putative phosphorylation site. Red one represented activated site and blue one represented inhibited site. PAS is Per-ARNTSim domain and has been shown to bind a diverse array of ligands. GAF is associated with signal sensing and ligand binding. HTH is the DNA binding domain, AAA^+^ is the central domain, PRD is the PTS regulatory domain, EIIA^Man^ is the mannose transporter enzyme IIA component domain, and EIIB^Gat^ is the galactitol transporter enzyme IIB component domain. **(B)** Gene organization of the *cel* gene cluster in *Bt* HD73. Open arrows represent ORFs. The bent arrow indicates the promoter upstream of *celA*. The nucleotide sequence represents the *celA* upstream region. The transcriptional start site (TSS) is marked in red and represented by a single solid underline and asterisks. The −24/−12 sequences are marked in blue and double-underlined. The putative CelR binding sites are marked in red and indicated as UAS1, UAS2, and UAS3. The putative CcpA binding site is marked in green and indicated as CRE.

Bacterial sigma factors are required for promoter recognition and transcription initiation by the bacterial RNA polymerase. Based on their function and mechanism, sigma factors fall into two broad families with no sequence homology: the more common Sigma70 family and the rarer Sigma54 family (Siegel and Wemmer, 2016; Zhang et al., 2016). The Sigma70 factor recognizes the typical −10/−35 promoter sequences. In contrast, Sigma54 directs the binding of polymerase to strongly conserved −12/−24 promoters, and the activation of Sigma54-RNA polymerase employs specialized bacterial enhancer-binding proteins (EBPs) whose activating function requires nucleotide hydrolysis (Buck et al., 2000; Danson et al., 2019). The EBPs are modular proteins and usually have three domains (Bush and Dixon, 2012). The N-terminal regulatory domain has a role in signal perception and modulates the activity of the EBPs. The central AAA^+^ domain is responsible for ATP hydrolysis. The C-terminal DNA binding domain contains a helix-turn-helix (HTH) motif (Bush and Dixon, 2012). For example, GabR and AcoR in *B. thuringiensis* are Sigma54-dependent activators that have three typical domains (Figure 1A). Their N-terminal regulatory domain, PAS domain in GabR and GAF domain in AcoR, play roles in the GABA-inducible transcription of *gabT* and acetoin-inducible transcription of *aco* operon. Their HTH motif is responsible for binding with *gabT* promoter and *acoA* promoter (Peng et al., 2014, 2020). In addition, there is a class of EBP that includes two regulatory domains (PRD) of the phosphoenolpyruvate (PEP): carbohydrate phosphotransferase system (PTS), which transports sugars, polyols, and other sugar derivatives (Deutscher et al., 2006).

The basic mechanisms of PRD-containing regulators are relatively well established in a number of gram positive bacteria, although individual species and proteins may differ in details. There are two types of PRD-domain containing transcriptional activators (Galinier and Deutscher, 2017). Each regulator type exhibits a priority for specific PTS families: MtlR/LicR-type regulators for PTSs of the fructose/mannitol, lactose/cellobiose, and galacticol families; LevR-like proteins for PTSs of the mannose family. Both MtlR/LicR-type transcriptional activators and LevR-type regulators contain an N-terminal DNA-binding domain and four regulatory domains, although their order and type of PTS domains are slightly different. The two PRDs usually present in MtlR/LicR-type transcriptional activators are preceded by an Mga-like domain and followed by two domains, which resemble EIIB^Gat^ and EIIA^Mtl/Fru^ PTS domains. Two PRD domains separate by EIIA^Man^ and EIIB^Gat^ PTS domains in LevR-type regulators. However, the most important difference is the insertion of a domain between the DNA-binding sequence and PRD1, which resembles the central domain in enhancer binding proteins.

Because of the central domain, LevR-like proteins activate the transcription from Sigma54-dependent promoters by binding to an upstream activating sequence (UAS). Only a few of the tasks of LevR-like proteins have been investigated. In *Bacillus subtilis*, the expression of the levanase (*lev*) operon is controlled by Sigma54, and the LevR activator (Figure 1A), which is a regulatory protein containing two C-terminal PRD domains and EIIA^Man^- and EIIB^Gat^-like domains inserted between the complete PRD1 and a truncated PRD2 (Martin-Verstraete et al., 1992, 1998). The LevR protein specifically binds to the UAS at −125 bases upstream from the transcriptional start site of the *lev* operon (Martin-Verstraete et al., 1994).

The activity of LevR is regulated by two PEP-dependent phosphorylation reactions catalyzed by the PTS system. The first phosphorylation at His-869 is catalyzed by P ~ EIIB^Lev^ and inhibits LevR activity. In the absence of fructose or mannose, EIIB^Lev^ is present mainly in its phosphorylated form. This allows the phosphorylation and inactivation of LevR by P ~ EIIB^Lev^, and the *lev* operon is poorly expressed. In contrast, in the presence of fructose, P ~ EIIB^Lev^ transfers its phosphate group mainly to the incoming sugar, leading to dephosphorylation and activation of LevR, which leads to the induction of the *lev* operon The second phosphorylation at His-585 catalyzed by P ~ His-HPr and stimulates LevR activity, which is presumed to play a role in carbon catabolite repression (CCR). It competes with the sugar-specific enzyme II of the PTS for the common phosphoryl donor P-His-HPr (Martin-Verstraete et al., 1998; Galinier and Deutscher, 2017).

*Listeria monocytogenes* contains only two LevR-like PRD-containing EBPs, i.e., ManR and CelR. ManR regulates the expression of the *man* operon that encodes the glucose/mannose PTS permease, which is transcribed from Sigma54-dependent promoters (Stoll and Goebel, 2010; Ake et al., 2011; Zebre et al., 2015). Another LevR-like transcription regulator CelR was identified, which was originally called LacR (Dalet et al., 2003), and it activates the expression of the cellobiose-induced PTS operons (Cao et al., 2019). In an earlier report, a similar functional protein CelR was also found in *Clostridium acetobutylicum* (Nie et al., 2016). The *cel* operon involved in cellobiose utilization is directly regulated by CelR and Sigma54 in *C. acetobutylicum*. CelR has an ATPase activity, which is strongly stimulated by the presence of DNA containing CelR-binding sites. CelR is regulated by PTS-mediated phosphorylation at His-551 and His-829, which exerts a positive and inhibitory effect on the CelR activity, respectively (Nie et al., 2016). In addition, the transcriptional regulons of about 50 PRD-containing EBPs in diverse *Firmicutes* species were reconstructed using a comparative genomic approach, analyzing genes associated with the utilization of β-glucosides, fructose/levan, mannose/glucose, pentitols, and glucosamine/fructosamine (Nie et al., 2016).

*Bacillus thuringiensis* (*Bt*) is a spore-forming gram-positive bacterium that forms parasporal crystals during sporulation that are toxic to a wide variety of insect larvae (Schnepf et al., 1998; Peng et al., 2020). *Bt* has been widely used as a biological pesticide. In our previous study, we identified 16 operons containing 47 genes whose transcription is controlled by Sigma54 (Peng et al., 2015). Eight Sigma54-dependent transcriptional EBPs were found in the genome of *B. thuringiensis* subsp. *kurstaki* strain HD73 (*Bt* HD73), and they regulated nine Sigma54-dependent promoters, which are involved in γ-aminobutyric acid shunt (Zhu et al., 2010; Peng et al., 2014), L-lysine metabolic pathway (Zhang et al., 2014), sarcosine utilization (Peng et al., 2016), and acetoin catabolic pathway (Peng et al., 2020). Among these eight EBPs, CelR (named as LevR in previous study) is the only PRD-domain containing activator, which belongs to the LevR-type family of regulators. CelR regulates *cel* operon, which is involved in cellobiose utilization in *Bt* HD73 (Peng et al., 2015).

In this study, we focused on transcriptional regulation of the *cel* operon, which participates in cellobiose utilization in *Bt* HD73. We found that transcription of the *cel* operon is induced by cellobiose and repressed by glucose. It contains a − 12/−24 promoter recognized by Sigma 54. The PRD-containing positive regulator CelR functions as an enhancer binding protein for Sigma54. In contrast, CelR and glucose-PTS work together to regulate glucose-mediated catabolite repression, while the catabolite control protein A (CcpA) appears capable of modulating this activity.

## 2 Results

### 2.1 Determination of the transcriptional units and transcriptional start site in the *cel* locus

The nucleotide sequence of the *cel* locus (4,214 bp) of *Bt* HD73 comprises five open reading frames (ORFs) and encodes five proteins, which have been annotated as the PTS system enzymes, including a cellobiose-specific IIB component (*celA*, HD73_5614), cellobiose-specific IIC component (*celB*, HD73_5613), cellobiose-specific IIA component (*celC*, HD73_5612), a 6-phospho-beta-glucosidase (*celD*, HD73_5611), and an unknown function protein (*celE*, HD73_5610) (Figure 1B). The gene encoding the Sigma54-dependent transcriptional activator (*celR*, HD73_5607) is located two genes-downstream of these five genes. The transcriptional units in the *cel* locus were determined using reverse transcription (RT)-PCR. The RT products of the genes flanking the genes encoding the five ORFs in the *cel* locus were amplified (Supplementary Figure S1A); however, no positive signals were detected for amplicons either upstream of *celA* or downstream of *celE* (Supplementary Figure S1A). These results suggest that the five genes of the *cel* locus form one transcriptional unit, *celABCDE*. To determine the transcriptional start site (TSS) of the *cel* operon, 5′-RACE analysis was performed as described in the material and methods section. Based on the sequence alignment of 20 random clones, a G residue located 55 bp upstream from the *celA* start codon was identified (Supplementary Figure S1B). According to promoter sequence alignment analysis on DBTBS^1^ (Sierro et al., 2008), a putative Sigma54-binding sequence GGCACGCTAATTGC (the double-underline indicates −12/−24 region of consensus sequence) was located 13 bp upstream of the *celA* TSS (Figure 1B). A putative CcpA binding site (CRE, catabolite responsive element) TGAAAGCGTTAACA, which was consistent with the consensus sequence, TGWAANCGNTNWCA (Weickert and Chambliss, 1990), was located upstream of the Sigma54-binding site (Supplementary Figure S2).

CelR (HD73_5607) is a putative PTS regulation domain-containing, Sigma54-dependent transcriptional activator, which includes an N-terminal DNA-binding domain containing a helix-turn-helix (HTH) motif, a central AAA^+^ domain, a mannose transporter enzyme IIA component domain (EIIA^Man^), a galactitol transporter enzyme IIB component domain (EIIB^Gat^), and two PTS regulation domains (PRDs) in the C-terminal region, unlike GabR or AcoR (Figure 1A) typical bacterial EBPs. It shares 32.7, 42.1, and 31.3% amino acid sequence identity with the homologs LevR (BSU27080) in *B. subtilis*, CelR (CA_C0382) in *C. acetobutylicum*, and ManR (lmo0785) in *L. monocytogenes*, respectively (Figure 1A).

### 2.2 Transcription of the *cel* operon is induced by cellobiose and regulated by CelR

**Figure 2:**
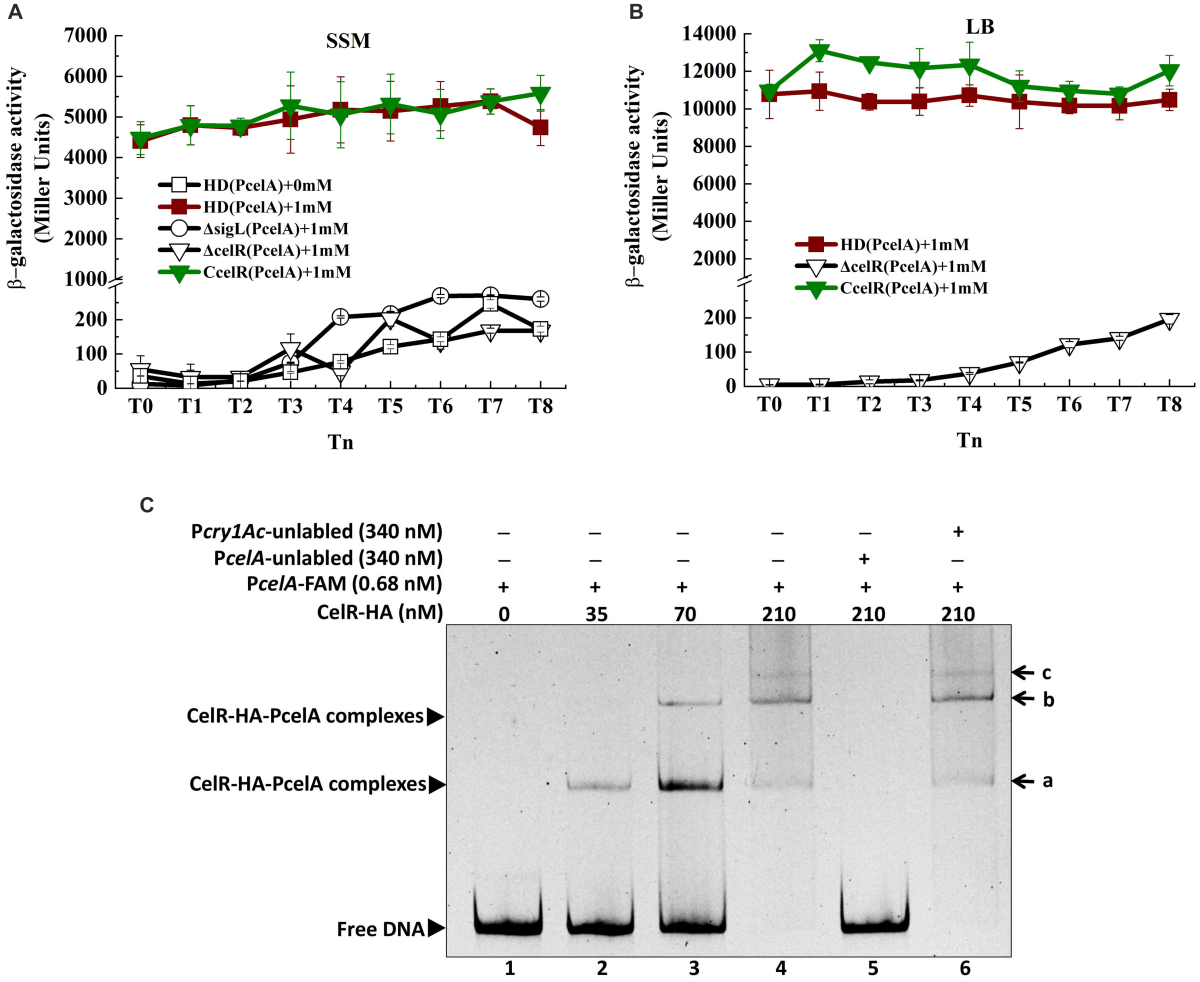
Transcription of *cel* operon is induced by cellobiose and regulated by CelR **(A)** β-Galactosidase activity of the *celA* promoter with a *lacZ* fusion (P*celA*) in wild-type HD73 with cellobiose (■) and without cellobiose (□) and in *sigL* (○) and *celR* mutants (▽) and the *celR* complemented strain (▼) with cellobiose in SSM medium. **(B)** β-Galactosidase activity of the *celA* promoter with a *lacZ* fusion (P*celA*) in wild-type HD73 (■), *celR* mutants (▽), and the *celR* complemented strain (▼) with 1 mM cellobiose in LB medium. **(C)** Electrophoretic mobility shift assay (EMSA) of CelR-HA protein with the *celA* promoter region. Lane 1, FAM-labeled P*celA* probe (346 bp, 0.68 nM); lanes 2–4, different concentrations of CelR-HA incubated with the probe; lane 5, incubation of 500-fold greater unlabeled P*celA* probe mixed with the labeled P*celA* probe and 210 nM CelR-HA; lane 6, incubation of 500-fold greater unlabeled P*cry1Ac* probe mixed with the labeled P*celA* probe and 210 nM CelR-HA. a, b, and c indicate different binding complexes.

A putative Sigma54-binding sequence and CelR binding sequences were located upstream of the *celA* TSS (Figure 1B). Alignment of these sequences is shown in Supplementary Figure S2. To identify whether transcription of the *cel* gene cluster is regulated by Sigma54 and CelR, the *celA* promoter (P*celA*) was ligated into the shuttle vector pHT304-18Z, which contains a promoter-less *lacZ* gene, as described in Material and methods section. The recombinant plasmid p18Z-P*celA* was introduced into *Bt* HD73 and the mutants. The expression of P*celA* in *Bt* HD73 wild-type, *sigL* (encodes Sigma54) and *celR* mutants, and the complemented strain of the *celR* mutant (CcelR) were investigated. The high level expression promoter P4468, which is related to isoleucine and valine degradation pathway (Peng et al., 2015), was used to direct the expression of CelR protein to complement the *celR* mutant. The strains were cultured in the medium containing 1 mM cellobiose, and samples were collected from T_0_ to T_8_ (T_0_ marks the end of the exponential phase, Tn represents n hours after the end of the exponential phase). β-Galactosidase assays showed that P*celA* transcriptional activity significantly increased and maintained a high expression level in HD73 wild-type strain grown in SSM supplemented with 1 mM cellobiose compared to that grown in SSM without cellobiose from T_0_ to T_8_ (Figure 2A). This indicated that P*celA* transcription is induced by cellobiose. However, P*celA* transcriptional activities were almost abolished in the Δ*sigL* and Δ*celR* mutants in the presence of cellobiose (Figure 2A). The induced activity of P*celA* was recovered in the *celR* complemented strain (CcelR) (Figure 2A). The growth rate of Δ*sigL* and Δ*celR* mutants did not significantly differ from that of the wild-type in SSM with or without cellobiose (Supplementary Figure S3). The cellobiose-induced activity of P*celA* was maintained a high expression level from T_0_ to T_8_ (Figure 2B). These results indicated that transcription of the *celABCDE* operon is induced by cellobiose, controlled by Sigma54, and positively regulated by CelR. It is interesting to note that *celABCDE* operon thought to be primarily involved in cellobiose utilization, is strongly expressed in stationary phase.

To confirm whether CelR can bind to the promoter of *celA* operon, CelR protein was expressed in *E. coli*. However, despite denaturation and renaturation, the CelR protein is insoluble, making its purification challenging. Therefore, the HTH-AAA^+^ (HA) domain in CelR was expressed with the N-terminal hexahistidine-tag and purified using a nickel-chelating affinity column, as described in the material and methods section. Electrophoretic mobility shift assay (EMSA) was performed to examine the ability of CelR-HA to bind directly to the P*celA* (346 bp) promoter. The shifted bands of PcelA-CelR-HA complexes were observed with increasing amounts of CelR-HA (Figure 2C). DNA fragment at 0.68 nM was completely shifted with 210 nM CelR-HA. At lower concentrations of CelR-HA, two shifted bands (labeled as a and b in Figure 2C) were observed, and the third band (labeled as c in Figure 2C) was visible at CelR-HA concentration at 210 nM. These results suggested that there may be three CelR-binding sites on the *celA* promoter. A 500-fold molar excess of the unlabeled P*celA* probe competed with the labeled P*celA* probe, indicating specific binding (Figure 2C, lane 5). However, a 500-fold molar excess of the unlabeled P*cry1Ac* probe (the negative control) (Yang et al., 2012) could not compete with the labeled P*celA* probe. It confirmed that CelR-HA did not bind to the *cry1Ac* promoter, which lacks a putative CelR-binding site (Figure 2C, lane 6). These results indicate that CelR-HA recognizes and binds specifically to sequences in the *celA* promoter fragment.

**Figure 3:**
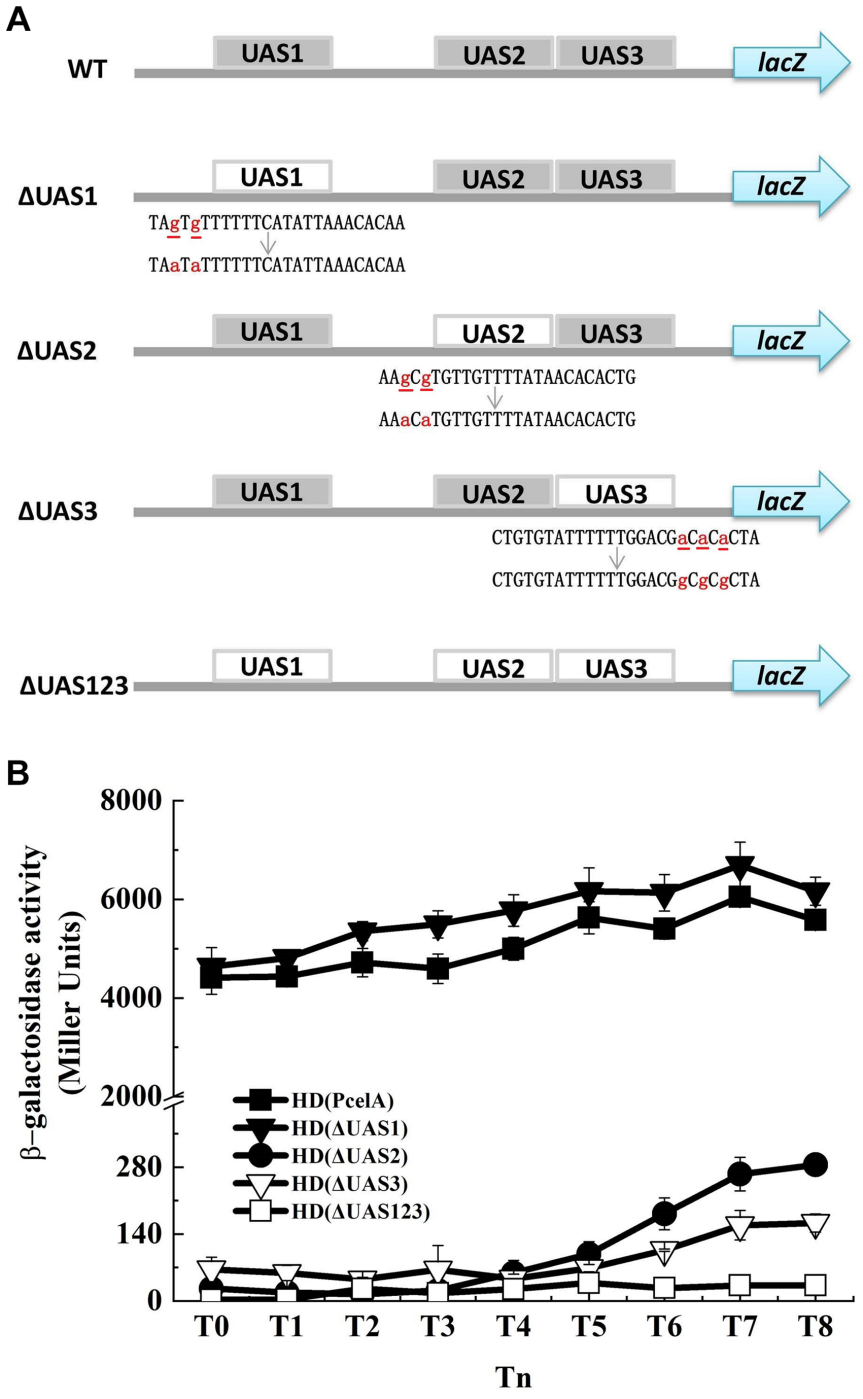
Mutation of putative CelR binding sites has effect on P*celA*. **(A)** WT is the wild-type P*celA* containing three upstream activity sites (UAS1, UAS2, and UAS3). ΔUAS1, ΔUAS2, and ΔUAS3 represent P*celA* containing the UAS1, UAS2, and UAS3 mutated fragments, respectively. ΔUAS123 is P*celA* containing the triple UAS mutant fragments. The small letters with underline represent original bases, and the small letters in red represent mutant bases. *lacZ* gene is a reporter gene located on the plasmid pHT304-18Z and encodes β-galactosidase. **(B)** β-Galactosidase activity of the *celA* promoter (■), ΔUAS1 (▼), ΔUAS2 (●), ΔUAS3 (▽), andΔUAS123 (□) with *lacZ* fusions in wild-type HD73 exposed to 1 mM cellobiose.

Three CelR-binding sites (UAS, upstream activity sites) were predicted in the promoter region of the *cel* operon according to the conserved CelR-binding DNA motif (Nie et al., 2016) (labeled as UAS1, UAS2, and UAS3 in Figure 1B). To determine whether these UAS sites are involved in the transcription of the *cel* operon, we constructed a *cel* promoter containing individual or triple UAS mutated fragments fused with the *lacZ* reporter gene and expressed in the HD73 wild type strain (Figure 3A). β-Galactosidase assay showed that mutation in UAS1 had no significant effect on the activity of *cel* promoter. However, mutations in UAS2 and UAS3 reduced the transcriptional activity of the wild-type *cel* promoter in the presence of cellobiose, and the transcriptional activity of ΔUAS123 was almost abolished compared with that of wild-type P*celA* (Figure 3B). These results indicated that UAS2 and UAS3 sites contribute to the transcription of the *cel* operon *in vivo*.

### 2.3 Point mutations of the EIIA^Man^ and EIIB^Gat^ domains and PRD affect transcription of the *cel* operon

**Figure 4:**
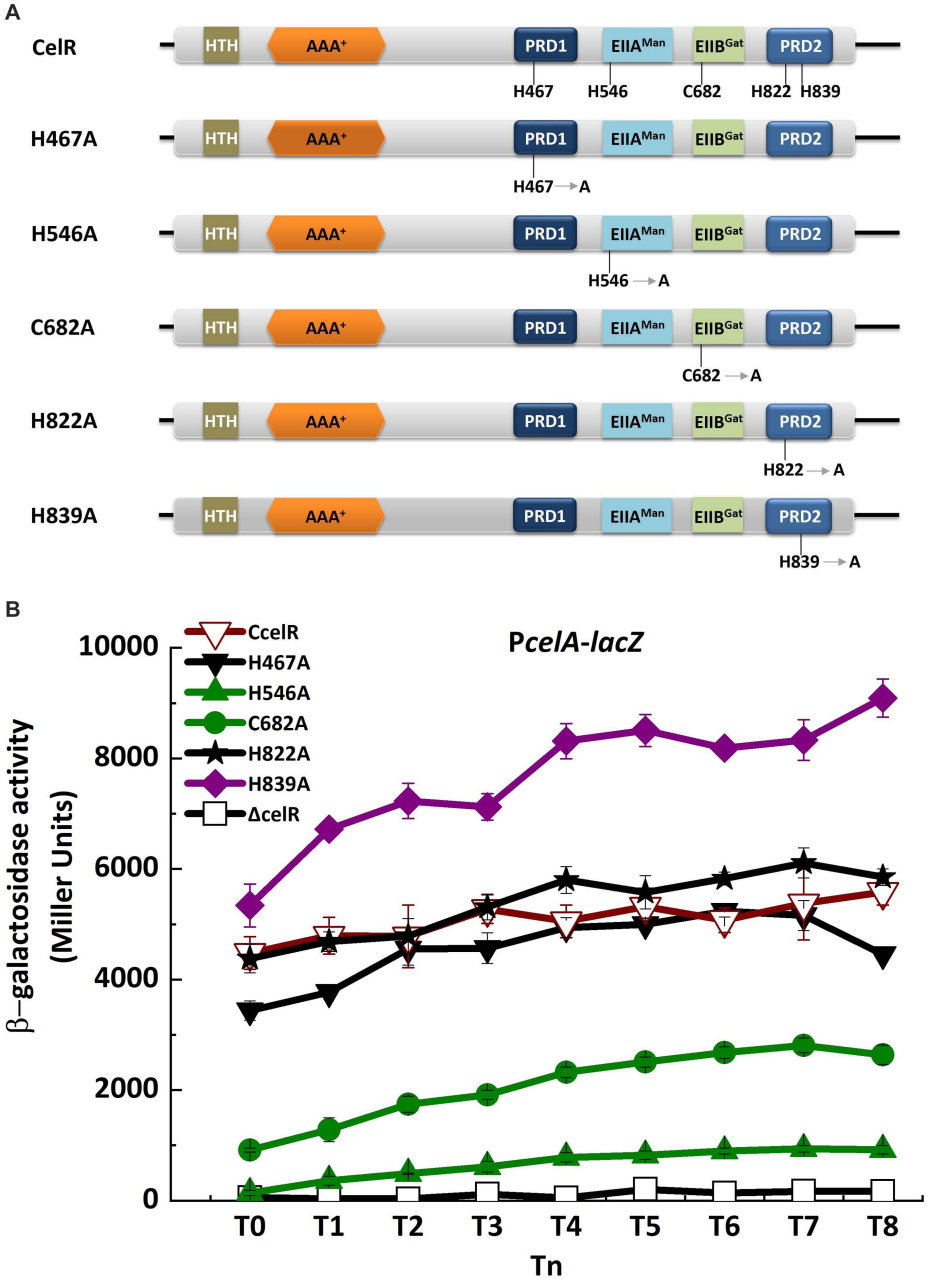
Point mutations of EIIA^Man^ and EIIB^Gat^ domains and PRD affect the transcription of *cel* operon. **(A)** Domain organizations of CelR containing histidyl or cysteine residues point mutant fragments. H467A is the histidyl residue at position 467 replaced by alanine. H546A is the histidyl residue at position 546 replaced by alanine. C682A is the cysteine residue at position 682 replaced by alanine. H822A is the histidyl residue at position 822 replaced by alanine. H839A is the histidyl residue at position 839 replaced by alanine. **(B)** β-Galactosidase activity of the *celA* promoter with a *lacZ* fusion (P*celA*) in *celR* mutant (□), *celR* complemented strain (▽), H467A mutant (▼), H546A mutant (▲), C682A mutant (●), H822A mutant (★), and H839A mutant (◆) with 1 mM cellobiose in SSM at T_0_ to T_8_.

Two PRDs are present in the C-terminal region of CelR, which are separated by the EIIA^Man^ and EIIB^Gat^ domains (Figure 1A). This characteristic is similar to that of LevR in *B. subtilis* and CelR in *C. acetobutylicum*. LevR in *B. subtilis* is regulated by PTS-mediated phosphorylation at the histidyl residues His-585 and His-869 (Martin-Verstraete et al., 1998). The *C. acetobutylicum* CelR is also regulated by PTS-mediated phosphorylation at His-551 and His-829, which exert a positive and inhibitory effect, respectively, on CelR activity (Nie et al., 2016). The equivalents of these two histidyl residues in *Bt* CelR are His-546 on the EIIA^Man^ domain and His-822 on PRD2, as determined by sequence alignment (Figure 4A; Supplementary Figure S4). To determine the role of the histidyl residues in the transcription of *cel* operon, we constructed a CelR complemented strain and two point mutants in which His-546 and His-822 were replaced with alanine (The experimental approach is illustrated in Supplementary Figure S4). The high level expression promoter P4468 was used to direct the expression of CelR (as in Figure 2A) and of CelR protein with mutations His546A and His822A. Next, we analyzed the capacity of the point mutants to induce transcription from the *cel* promoter compared with that of the CelR complemented strain. β-Galactosidase activity showed that the transcriptional activity of P*celA* was sharply decreased in the *celR* mutant compared to that in the wild-type strain in the presence of cellobiose (Figure 2A). The mutant CelR H822A fully complemented the *celR* deletion mutation as compared with wild-type CelR (Figure 4B), whereas the expression of P*celA* in the strain carrying CelR H546A was very low and similar to that in the *celR* mutant strain. According to CelR sequence alignment (Supplementary Figure S5), the other three putative phosphorylation sites, i.e., His-467, Cys-682, and His-839, were found in PRD1, the EIIB^Gat^ domain, and PRD2, respectively. To determine whether these residues affect transcription of the *cel* operon, His-467, Cys-682, and His-839 were mutated to alanine, respectively. β-Galactosidase assay showed that the transcriptional activity of P*celA* was increased in the H839A mutant, decreased in the C682A mutant, and had no significant difference in the H467A compared to that in the complemented strain CcelR in the presence of cellobiose (Figure 4B). All these results indicated that His-546 and Cys-682 exert a positive effect, His-839 exerts an inhibitory effect, and His-467 and His-822 have no effect on the CelR activity.

### 2.4 Transcription of *cel* operon is repressed by glucose and regulated by CcpA

**Figure 5:**
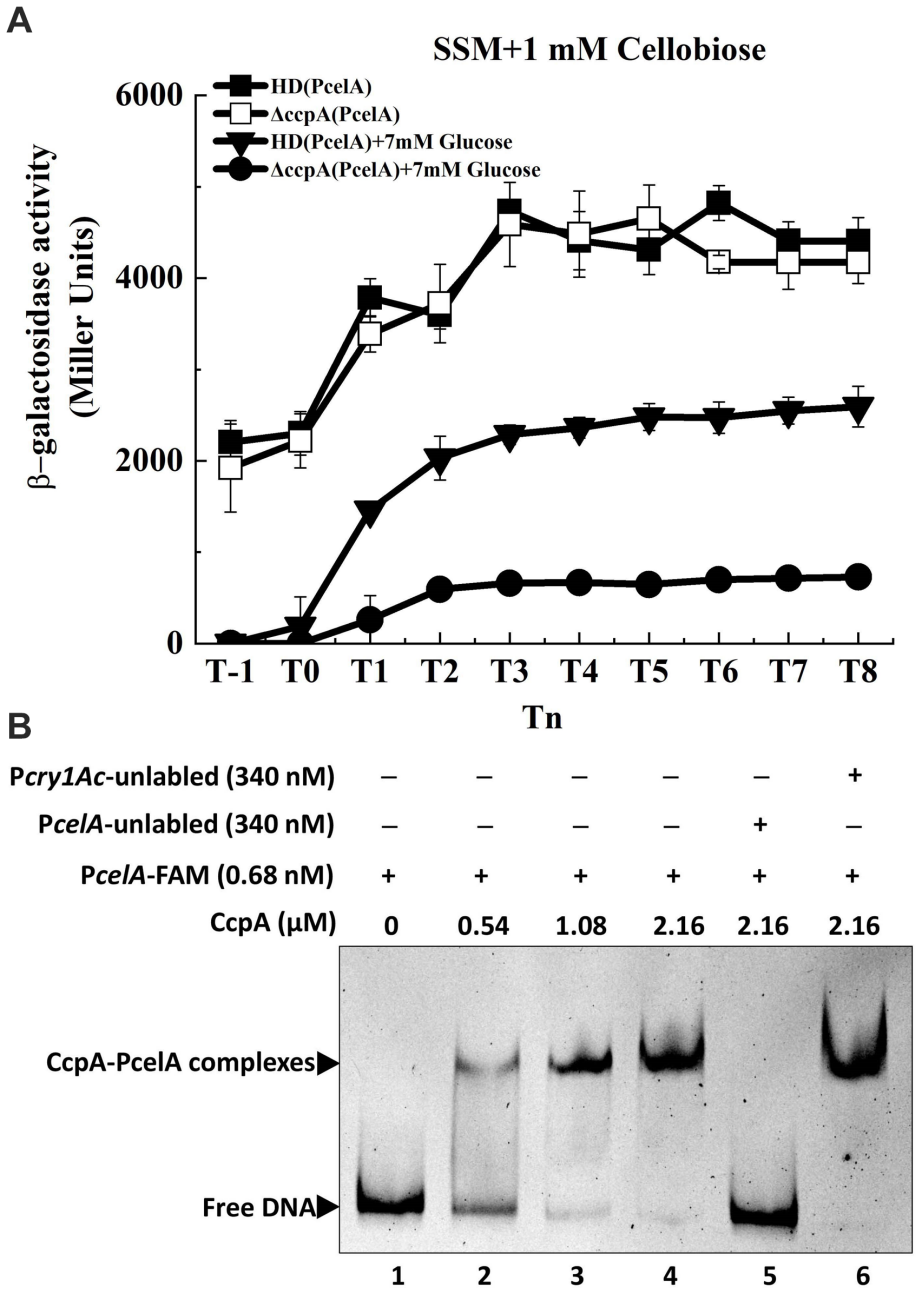
Transcription of *cel* operon is repressed by glucose and regulated by CcpA. **(A)** β-Galactosidase activity of the *celA* promoter with a *lacZ* fusion (P*celA*) in wild-type HD73 with cellobiose (■) or with both cellobiose and glucose (▼) and in *ccpA* mutant with cellobiose (□) or with both cellobiose and glucose (●) from T_−1_ to T_8_. **(B)** Electrophoretic mobility shift assay (EMSA) of CcpA protein with the *celA* promoter region. Lane 1, FAM-labeled P*celA* probe (346 bp, 0.68 nM); lanes 2–4, different concentrations of CcpA incubated with the probe; lane 5, incubation of 500-fold greater unlabeled P*celA* probe mixed with the labeled P*celA* probe and 2.16 μM CcpA; lane 6, incubation of 500-fold greater unlabeled P*cry1Ac* probe mixed with the labeled P*celA* probe and 2.16 μM CcpA.

Carbon catabolite repression (CCR) occurs when bacteria are exposed to two or more carbon sources, and one of them is preferentially utilized (frequently glucose). Most PTS-mediated CCR mechanisms respond to the phosphorylation level of a PTS protein, which is controlled by the metabolic state of the cell (Deutscher, 2008). To determine whether glucose or CcpA affects transcription of the *cel* operon in *Bt*, the expression of P*celA* in wild-type and *ccpA* mutant were investigated in SSM with 7 mM glucose. The strains were cultured in glucose-containing SSM until the mid-exponential phase (T_−1_). 1 mM cellobiose was added at T_−1_, and samples were collected 1-h intervals after induction. β-Galactosidase assay showed that transcriptional activity of the *celA* promoter (P*celA*) in the wild-type strain HD73 much lower in SSM supplemented with 7 mM glucose and 1 mM cellobiose, compared to that with cellobiose alone (Figure 5A). In the presence of 7 mM glucose and 1 mM cellobiose, P*celA* activity was significantly lower from T-_1_ to T8 in the *ccpA* mutant compared to that in the wild-type strain (Figure 5A). However, the cellobiose-induced transcriptional activity of P*celA* showed no significant difference between the wild-type strain and *ccpA* mutant in the absence of glucose (Figure 5A). The growth rate of Δ*ccpA* mutant was not significantly different from that of wild-type in SSM with or without cellobiose (Supplementary Figure S3). These results demonstrated that glucose repressed the cellobiose-induced transcription of P*celA*, which could be positively regulated by CcpA in the presence of glucose, or indirectly by the effect of CcpA exerted on the expression of certain glucose-PTS.

A putative CcpA binding site (*cre*) was found in the P*celA* sequence (Figure 1B) by analyzing the promoter sequence using the DBTBS database (Sierro et al., 2008). To determine whether P*celA* is directly or indirectly regulated by CcpA, CcpA protein was purified and EMSA experiments were performed. As shown in Figure 5B, the shifted bands of PcelA-CcpA complexes were observed with increasing amounts of CcpA and 0.68 nM DNA fragment which was nearly completely shifted by 2.16 μM CcpA. A 500-fold molar excess of the unlabeled P*celA* probe competed with the labeled P*celA* probe, indicating specific binding (Figure 5B, lane 5), while a 500-fold molar excess of the unlabeled P*cry1Ac* probe (the negative control) could not compete with the labeled P*celA* probe (Figure 5B, lane 6). These results indicate that CcpA recognizes and specifically binds to sequences within the *celA* promoter fragment.

### 2.5 The *cel* operon is responsible for cellobiose utilization

**Figure 6:**
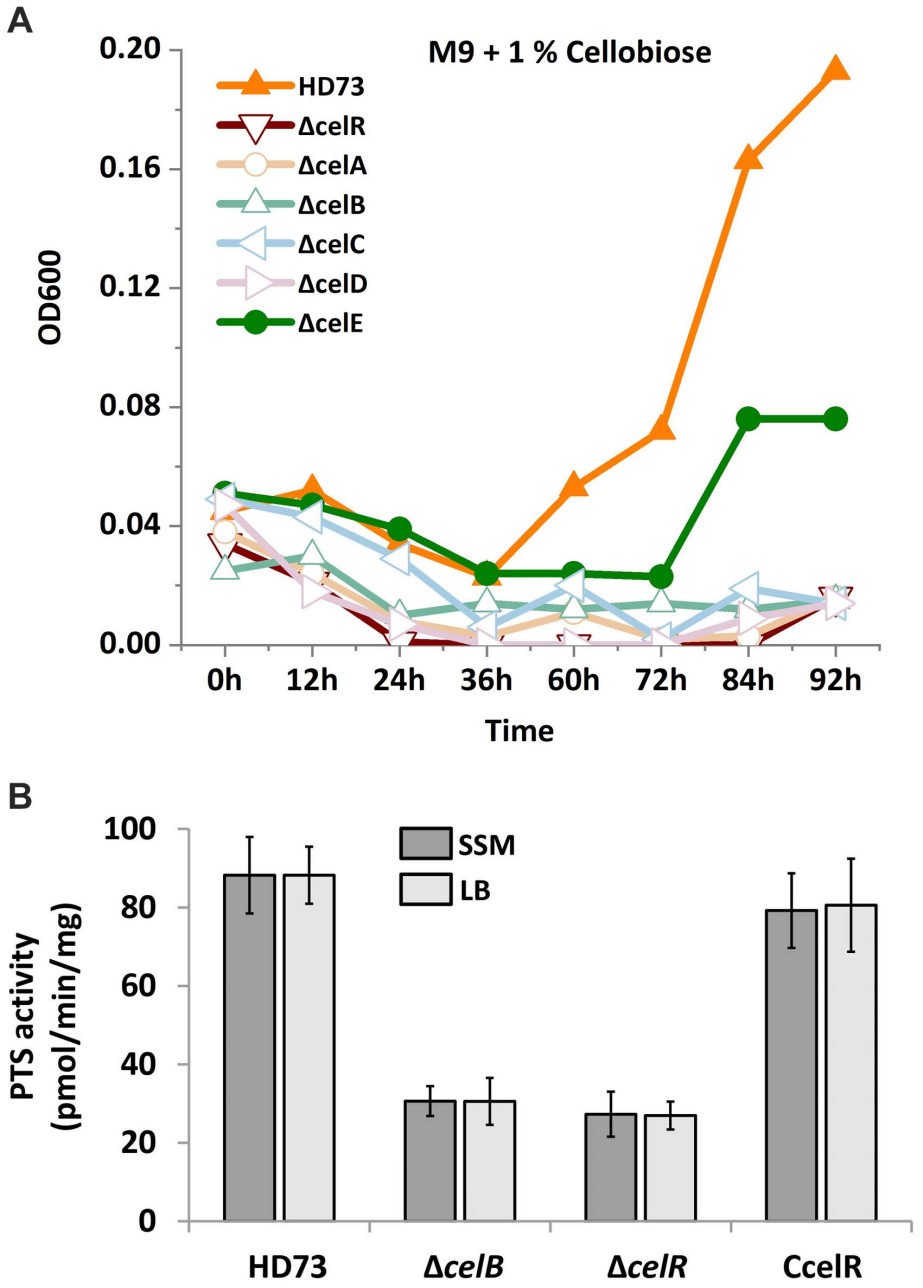
Cellobiose utilization and phosphotransferase system (PTS) activities in the *cel* mutants. **(A)** Growth curve of Bt HD73 and *cel* mutants. The cells were cultured in the M9 medium which was added 1% cellobiose (M/V) as the sole carbon at 30°C and 220 rpm. Next, OD600 was measured at 3 h intervals. The *y* axis represents OD600 in different strains. The *x* axis represents different time. **(B)** PTS activities. The strains were cultured in LB and SSM medium supplemented with cellobiose until mid-exponential phase. The samples were resuspended in 0.1 M PBS buffer and mixed with the reaction solution after measuring the dry weight. The PTS activity was monitored by measuring the absorbance at 340 nm. The *y*-axis represents PTS activity in different strains. The *x*-axis represents wild-type HD73 and different mutants. The reported value is the mean value from at least three independent assays.

Proteins encoded by the *cel* operon are homologous to known functional proteins in other bacteria (Nie et al., 2016; Cao et al., 2019). Hence, *cel* operon mutants were constructed to examine whether the utilization of cellobiose and PTS activity was affected. Growth in the presence of cellobiose was monitored, and the PTS activity assay was performed. According to the growth curve (Figure 6A), HD73 wild-type strain began to grow after 36 h in the M9 medium with 1% cellobiose as the sole carbon source compared to that in the absence of cellobiose. However, none of mutants could grow in the M9 medium in the presence of cellobiose, except the *celE* mutant, which demonstrated slow growth. While, HD73 wild-type strain and all the mutants could grow in the M9 medium with 1% glycerol as the sole carbon source (Supplementary Figure S6). In order to determine the sugar-specific function of the permease component, we performed PTS activity assay in WT, *celB* mutant, *celR* mutant, and *celR* complementary strain. The strains were cultured in LB and SSM medium supplemented with cellobiose until mid-exponential phase, and cells were harvested and the pellets were resuspended in 0.1 M PBS buffer. The PEP-dependent PTS activity assay is described in Material and methods section. The PTS activities of *celB* and *celR* mutants were decreased compared with that of the wild-type strain and celR complementary strain (Figure 6B). These results indicated that the *cel* operon is responsible for cellobiose transport and utilization.

## 3 Discussion

The mechanism underlying the regulation of PRD-containing transcriptional factors is certainly the one most intertwined with the PTS (Galinier and Deutscher, 2017), and PRD-containing regulators are controlled by PTS-dependent phosphorylation (Stulke et al., 1998). Without an inducer, the phosphorylated EIIB component of the sugar permease donates its phosphate to a PRD, thereby inactivating the regulator. In the presence of the substrate, the regulator is dephosphorylated, and the phosphate is transferred to the sugar, resulting in the induction of the operon (Stulke et al., 1998). In the presence of an efficiently utilized carbon source, ATP-dependent phosphorylation of HPr at Ser-46 by HPr kinase inhibits phosphorylation by enzyme I; therefore, PRD-containing regulators cannot be stimulated and are inactive. Most regulators of this family contain two copies of PRD, which are functionally specialized in either induction or CcpA-independent CCR (Stulke et al., 1998; Galinier and Deutscher, 2017). In some LevR-like regulators of the order *Clostridiales*, the truncated PRD2 can be replaced with an EIIA^Mtl^-like domain (Deutscher et al., 2014). In *B. subtilis*, LevR contains the histidyl residues His-585 in EIIA^Man^ and His-869 in PRD2 domains. Phosphorylation at His-585 is catalyzed by P ~ His-HPr and stimulates LevR activity. Phosphorylation at His-869 is catalyzed by P ~ EIIB^Lev^ and inhibits LevR activity (Martin-Verstraete et al., 1998). The equivalents of these two histidyl residues in *C. acetobutylicum* CelR are His-551 and His-829. Replacement of His-551 with alanine resulted in the loss of stimulation of CelR activity, suggesting that the phosphorylation of His-551 exerts a positive effect on the CelR activity. However, the *celC* gene was expressed at a high level in the strain carrying the mutated protein CelR-H829A, regardless of the presence or absence of cellobiose. This suggested that phosphorylation of His-829 inhibits CelR activity in *C. acetobutylicum* (Nie et al., 2016).

A similar observation was reported in our study, showing that the replacement of His-546 on the EIIA^Man^ domain of CelR with alanine resulted in the loss of CelR activity. In contrast, mutation of His-822 on PRD2 did not affect the CelR activity. However, mutation of non-conserved His-839 on PRD2 enhanced the CelR activity. His-839 is only present in the majority *Bacillus* species except *B. subtilis* (Supplementary Figure S5).

In the presence of glucose, the induced transcription of *cel* operon was decreased. It may be because phosphorylation by P ∼ His-HPr at His-546 in the EIIA^Man^ domain was suppressed in *Bt*, leading to the inactivation of CelR. We assumed that the phosphorylation occurred at His-546 on EIIA^Man^, rather than His-822 on PRD2. In the presence of cellobiose, we speculated that PRD2 is dephosphorylated at His-839 as the phosphate is transferred to the cellobiose, resulting in transcription induction of the *cel* operon. Other putative phosphorylation sites, His-467 and Cys-682, were also found in the PRD1 and EIIB^Gat^ domains of CelR in *Bt*. Mutation of His-467 has no effect on CelR activity, and mutation of Cys-682 decreased CelR activity. The equivalents of these two residues are His-506 and Cys-718 in *B. subtilis* LevR, and His-468 and Cys-683 in *L. monocytogenes*. However, phosphorylation of EIIB^Gat^ in LevR-like regulators has not been reported, and its regulatory role remains unknown. Here, we provided evidence that Cys-682 on the EIIB^Gat^ domain stimulates CelR activity in *Bt*.

**Figure 7:**
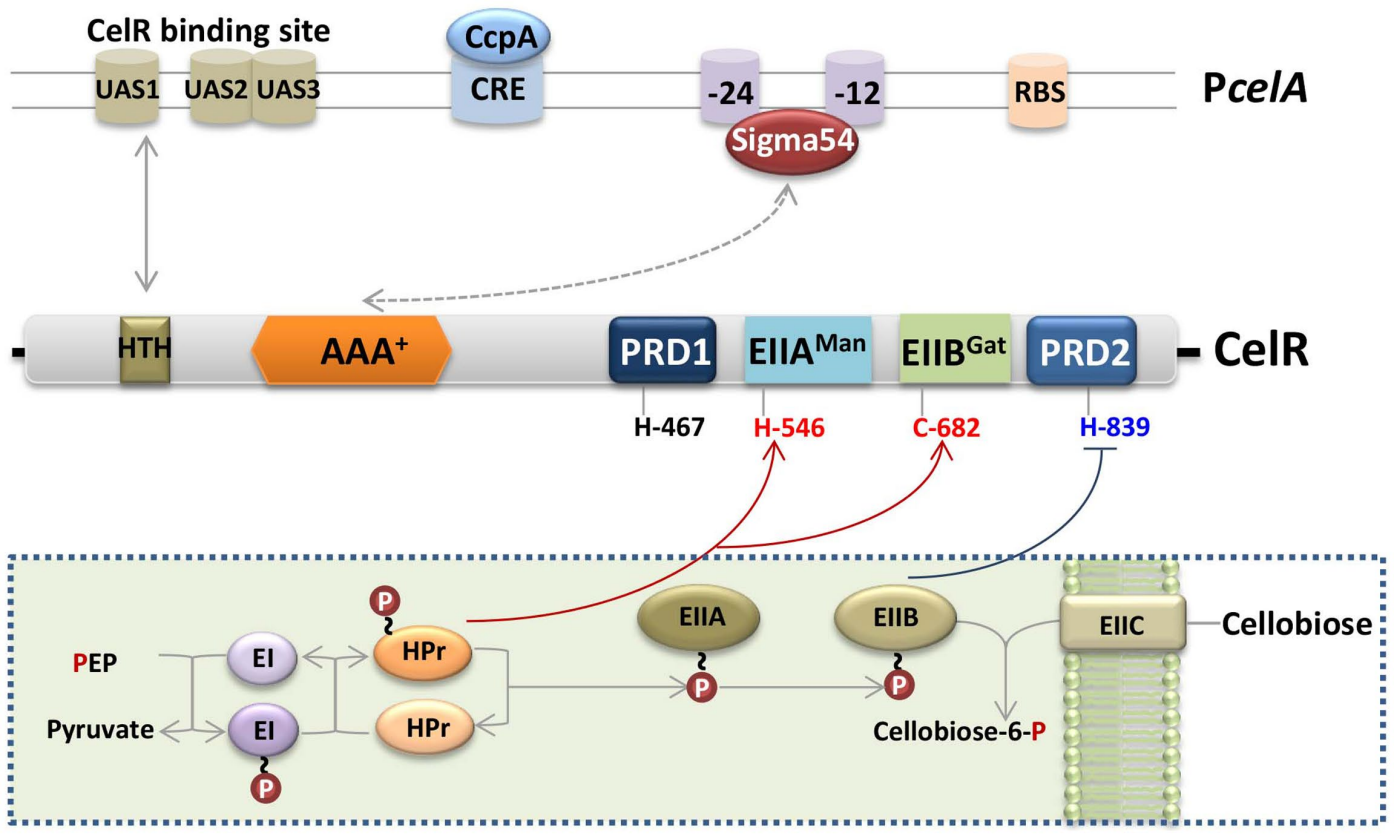
Schematic diagram of the regulatory model of the *cel* gene cluster in Bt. The *cel* operon encodes the phosphotransferase system (PTS), which is responsible for the transport and utilization of cellobiose. In the absence of cellobiose, EIIB^Cel^ (represented by a light brown oval) is present mainly in its phosphorylated form, resulting in the phosphorylation and inactivation of CelR by P ~ EIIB^Cel^, while the *cel* operon is poorly expressed. In contrast, in the presence of cellobiose, EIIC (represented by a light brown rounded square) transports extracellular cellobiose to the cytoplasm, and P ~ EIIB^Cel^ subsequently transfers its phosphate group (represented by a red circle) to cellobiose, leading to the dephosphorylation and activation of CelR. CelR activity is stimulated by phosphorylation at His-546 or Cys-682 and inhibited by phosphorylation at His-839. Simultaneously, the AAA^+^ domain of CelR interacts with Sigma54 (represented by a grey double-headed arrow and dotted line), the HTH domain of CelR (represented by a brown square) binds to the *cel* promoter (represented by a grey double-headed arrow), and the DNA-binding domain of Sigma54 binds to the conserved −24/−12 sites on the promoter, which co-leads the induction of the *cel* operon. Cellobiose-induced transcription of the *cel* operon was repressed by glucose via CcpA, and CcpA specifically bound to the *cre* sequences within the *cel* promoter fragment. Red lines with an arrow represent activation, and dark blue lines with horizontal lines represent inhibition. The box with dotted line indicated the hypothetical functions of *cel* operon.

*Bt*, *Bacillus cereus* (an opportunistic human pathogen), and *Bacillus anthracis* (the etiological agent of anthrax in mammals) are the members of the *B. cereus* group (Han et al., 2006). The *cel* gene clusters among *B. cereus* group bacteria share high sequence similarity and their organization is similar (Supplementary Figure S7). The domains of the PRD-containing Sigma54-dependent transcription activator CelR from *Bt* HD73 and four phosphorylation sites (His-467, His-546, Cys-682, and His-839) were conserved in the genomes of other species in *Bacillus cereus* group (Supplementary Figure S5). These results suggest that Sigma54 and CelR regulate the expression of the *cel* operon in these bacteria. Based on our findings, we propose a hypothetical schematic diagram of the transcription and regulation of the *cel* operon in *Bt* (Figure 7). In the absence of cellobiose, P ~ EIIA (CelC) transfers the phosphoryl group to its cognate EIIB (CelA), which is present mainly in its phosphorylated form, resulting in the phosphorylation and inactivation of CelR by P ~ EIIB (CelA), while the *cel* operon is expressed at a low level. In contrast, in the presence of cellobiose, EIIC (CelB) transports extracellular cellobiose to the cytoplasm, and P ~ EIIB subsequently transfers its phosphate group to cellobiose, leading to the dephosphorylation and activation of CelR. We propose that CelR activity is stimulated by phosphorylation at His-546 or Cys-682, and inhibited by phosphorylation at His-839. Simultaneously, the AAA^+^ domain of CelR interacts with Sigma54, the HTH domain of CelR binds to the *cel* promoter, and the DNA-binding domain of Sigma54 binds to the conserved −24/−12 sites on the promoter, which leads to the induction of the *cel* operon. However, in the presence of glucose and cellobiose, ATP-dependent phosphorylation of HPr occurs at Ser-46 by HPr kinase, inhibiting phosphorylation by EI. As a result, CelR cannot be phosphorylated at His-546 or Cys-682 and is inactivated, and the transcriptional activity of the *cel* operon is decreased. CcpA participates in the inhibitory effect of glucose on the *cel* promoter by binding to *cre* sequences within the *cel* promoter fragment.

Cellobiose utilization pathways have always been of particular interest due to their potential use for developing of alternative energy sources (Desvaux, 2006; Zeng and Burne, 2009). The *cel* gene cluster encodes the components of the cellobiose utilization pathway in many bacterial species (Parker and Hall, 1990; Lai and Ingram, 1993; Tobisch et al., 1997; Zeng and Burne, 2009; Shafeeq et al., 2011; Solopova et al., 2012; Wu et al., 2012; Nie et al., 2016; Cao et al., 2019; Hasan et al., 2021; Qu et al., 2021). However, their gene organization and transcriptional mechanisms can largely vary. For instance, in *Streptococcus mutans*, a six-gene locus (*celA*-*celB*-*celR*-*celC*-*Smu1452*-*celD*) in the genome encoded putative cellobiose-specific PTS enzymes and a regulator CelR and was organized into two transcriptional units from promoters that are proximal to *celA* and *celB* (Zeng and Burne, 2009). Their promoters require CelR for cellobiose-induced transcriptional activation, but read-through from the *celA* promoter contributes to the expression of the cellobiose transporter enzyme II component (EII^Cel^) genes, which are required for the transcriptional activation of the *cel* genes. Similarly, the *cel* locus, which is regulated by CelR, was also found in *S. pneumoniae* (Shafeeq et al., 2011). CelR in *S. mutans* and *S. pneumoniae* are not direct homologs of EBP PRD proteins but belong to the LicR type of PRD proteins. For both bacteria, CcpA plays a relatively minor direct role in the catabolite repression of the *cel* regulon, even though their promoters have the putative CcpA binding catabolite-responsive elements (*cre*) (Zeng and Burne, 2009; Shafeeq et al., 2011). However, in *Lactococcus lactis*, CcpA is involved in the transcriptional regulation of the promoter of the *cel* gene cluster, which encodes a putative cellobiose-specific PTS IIC component (Solopova et al., 2012). In *B. subtilis*, the expression of the *licBCAH* operon (the previous name is *cel*) is induced by the products of lichenan hydrolysis, i.e., lichenan and cellobiose, which are regulated by LicR (the previous name is CelR) and controlled via CCR (Tobisch et al., 1997).

In our study, *cel* operon consists of five genes. Three of them, *celA*, *celB*, and *celC*, encode PTS system enzymes. *celD* encodes a 6-phospho-beta-glucosidase. CelE is an unknown function protein, which shares 49% with CelC of *Bacillus stearothermophilus*. It is an YdjC-like protein, and possibly involved in the cleavage of cellobiose-phosphate (Lai and Ingram, 1993). Unlike the transcriptional regulation of the *cel* operons mentioned above, the promoter of the *cel* operon used in our study is a Sigma54-dependent promoter, and its transcription is regulated by the PRD-domain containing Sigma54-dependent activator CelR. Additionally, this promoter is positively regulated by CcpA in the presence of glucose. This regulation is similar to the regulations of the *cel* operon in *L. monocytogenes* (Cao et al., 2019) and *C. acetobutylicum* by CelR (Nie et al., 2016). CelR, in these two bacteria, is also a PRD-domain containing Sigma54-dependent activator, and transcription of their *cel* operons is controlled by Sigma54.

The *cel* gene clusters have different expression and regulation modes in different bacteria. The data presented here can improve our understanding of the transcription patterns of *cel* gene clusters, which will help us better to understand the utilization of cellobiose for bacterial growth.

## 4 Materials and methods

### 4.1 Bacterial strains, growth conditions, and DNA manipulation

The bacterial strains and plasmids used in this study are listed in Supplementary Table S1. *Escherichia coli* was cultured in Luria-Bertani (LB) medium with continuous shaking (220 rpm) at 37°C. *Bt* strains, including *Bt* strain HD73 (accession no. CP004069) (Liu et al., 2013) were cultured in Luria-Bertani (LB) medium or Schaeffer’s sporulation medium (SSM) (Schaeffer et al., 1965) with continuous shaking (220 rpm) at 30°C. Kanamycin (100 μg/mL), erythromycin (10 μg/mL), and ampicillin (100 μg/mL) were used for selection for *Bt* and *E. coli*. DNA manipulation was performed as previously described (Peng et al., 2016). Oligonucleotide primers are listed in Supplementary Table S2. All constructs were confirmed by sequencing (BGI, Beijing, China).

### 4.2 RT-PCR and 5′-RACE analysis

Total RNA was extracted at stage T_7_ from *Bt* cells grown in SSM as previously described (Du et al., 2012), and RT-PCR analysis was performed using primers RT-1 to RT-17. cDNA was synthesized and the transcriptional start site (TSS) of *celA* was determined using the SMARTer™ RACE cDNA amplification kit (Clontech, Mountain View, CA, USA), as previously described (Peng et al., 2016). Gene-specific primers (GSPs) and universal primer mix (UPM) were used to amplify the 5′ end of *celA* mRNA.

### 4.3 Construction of the *celR* and *celABCDE* mutants

The upstream fragment A (592 bp) and downstream fragment B (556 bp) of the *cel*R gene were amplified from *Bt* HD73 genomic DNA using PCR with the primers celRA-F/celRA-R and celRB-F/celRB-R. The kanamycin resistance cassette *kan* of 1,513 bp was amplified from pDG780 using PCR with the primers celR-kmF/celR-kmR. Fragment A, *kan*, and fragment B were ligated by overlapping PCR with the primers celRA-F and celRB-R. The resultant PCR product was digested, purified, and ligated into the vector pMAD (Arnaud et al., 2004). The recombinant plasmid pMADΔ*cel*R was introduced into *Bt* HD73, yielding the strain HD (pMADΔ*cel*R). The strain was grown overnight at 30°C in LB medium containing erythromycin and kanamycin. It was then transferred to LB medium containing only kanamycin at 39°C for 3 h. In that condition, the bacteria lost the pMAD vector and erythromycin resistance. Next, the cells were then plated on LB agar plates. The mutant strain Δ*celR* showing resistance to kanamycin but not to erythromycin was selected and verified using a PCR.

The DNA fragments corresponding to the upstream and downstream regions of the *celA* gene were amplified using PCR with celA-a/celA-b and celA-c/celA-d as primers, respectively. The corresponding DNA fragments were ligated via overlapping PCR with celA-a and celA-d as primers. The PCR product was then ligated into the vector pMAD. The recombinant plasmid pMADΔ*celA* was introduced into *Bt* HD73, and the strain HD (pMADΔ*cel*A) was obtained after screening for mutants as described above. The mutant strain, Δ*celA* without erythromycin resistance was selected, and verified using PCR.

The *celB*, *celC*, *celD* and *celE* mutants were constructed using a method similar to that for *celA* described above, except with the primers celB-a/celB-b/celB-c/celB-d, celC-a/celC-b/celC-c/celC-d, celD-a/celD-b/celD-c/celD-d, and celE-a/celE-b/celE-c/celE-d, respectively.

### 4.4 Construction of promoters with *lacZ* gene fusions

To determine the transcriptional activity of the *cel* operon, the wild-type promoter fragment of the *celA* gene (346 bp upstream of the start codon of *celA*) from *Bt* HD73 was amplified via PCR using the specific primers PcelA-F/PcelA-R. Oligonucleotide primers are listed in Supplementary Table S2, and restriction enzyme sites are underlined. The *Bam*HI/*Hin*dIII fragments of P*celA* were ligated into the shuttle vector pHT304-18Z, which contains a promoter-less *lacZ* gene (Agaisse and Lereclus, 1994). The recombinant plasmid p18Z-P*celA* was introduced into *Bt* HD73 and the Δ*sigL* (*sigL* encodes Sigma54) (Zhu et al., 2010), Δ*celR*, and Δ*ccpA* (Peng et al., 2020) mutants. The corresponding strains HD (P*celA*), Δ*sigL* (P*celA*), Δ*celR* (P*celA*), and Δ*ccpA* (P*celA*) were selected using erythromycin resistance and verified by PCR.

To determine the function of the CelR-binding site within the *celA* promoter, four mutated promoters (P*celA*-UAS1, P*celA*-UAS2, P*celA*-UAS3, and P*celA*-UAS123) containing mutations in one or all CelR binding sites were synthesized by the BGI group (Beijing, China). The sequences of the mutated sites are indicated in Figure 3A. The mutated promoters were amplified via PCR using the specific primers PcelA-F/PcelA-R, followed by digestion, purification, and ligation into the vector pHT304-18Z. The recombinant plasmids p18Z-UAS1, p18Z-UAS2, p18Z-UAS3, and p18Z-UAS123 were introduced into *Bt* HD73, producing the strains HD (ΔUAS1), HD (ΔUAS2), HD (ΔUAS3), and HD (ΔUAS123).

### 4.5 Genetic complementation of the *celR* mutant

High level expression promoter P4468 (Peng et al., 2015) was used to direct the expression of CelR. P4468 was amplified from HD73 genomic DNA using PCR with the primer pair P4468-1/P4468-2. *celR* fragment was amplified from HD73 genomic DNA using PCR with celR-F/celR-R as primers. The PCR products were purified and ligated into the plasmid pHT1618 (Lereclus and Arantes, 1992) using a seamless cloning kit. The resulting plasmid (pHT-CcelR) was introduced into the *Bt* mutant strain Δ*celR* (P*celA*), yielding the complemented strain C*celR* (P*celA*). This strain complements the *celR* mutant, allowing us to evaluate the expression of the *celA* promoter with a *lacZ* fusion.

### 4.6 Construction of point mutations in EIIA^Man^ and EIIB^Gat^ domains and PRD

To determine the function of His-467 on PRD1, His-822 and His-839 on PRD2 domain, His-546 on the EIIA^Man^ domain, and Cys-682 on the EIIB^Gat^ domain, five point mutants were constructed. The experimental approach is illustrated in Supplementary Figure S4. The upstream fragment (2,152 bp) and the downstream fragment (1,317 bp) of the *celR* gene containing the mutated site of His-467 were amplified using PCR with the plasmid pHT-CcelR as the template and the primers P4468-1/H1U-R and H1D-F/celR-R, respectively. CelR fragments (3,208 bp and 246 bp) with a mutation at His-822 were amplified using PCR with the plasmid pHT-CcelR as the template and P4468-1/H2U-R and H2D-F/celR-R as the primer pairs. CelR fragments (3,242 bp and 173 bp) with a mutation at His-839 were amplified using PCR with the plasmid pHT-CcelR as the template and P4468-1/H839U-R and H839D-F/celR-R as the primer pairs. CelR fragments (2,386 bp and 1,078 bp) with a mutation at His-546 were amplified using PCR with the plasmid pHT-CcelR as the template and P4468-1/H546U-R and H546D-F/celR-R as the primer pairs. CelR fragments (2,794 bp and 673 bp) with a mutation at Cys-682 were amplified using PCR with the plasmid pHT-CcelR as the template and P4468-1/H682U-R and H682D-F/celR-R as the primer pairs. Next, the PCR products were purified and ligated into the plasmid pHT1618 using a seamless cloning kit. The resulting plasmids (pHT-*celR*-H467A, pHT-*celR*-H822A, pHT-*celR*-H839A, pHT-*celR*-H546A, and pHT-*celR*-C682A) were introduced into the *Bt* mutant strain Δ*celR* (P*celA*), yielding the strains H467A (P*celA*), H822A (P*celA*), H839A (P*celA*), H546A (P*celA*), and C682A (P*celA*), respectively. These plasmids complement the *celR* mutant strain without the His467, His822, His839, His546, and Cys682 residues, respectively, allowing us to evaluate the expression of the *celA* promoter using *lacZ* fusion constructs.

### 4.7 Expression and purification of the HTH-AAA^+^ domain of CelR

The fragment (from nucleotides 1 to 759 after the ATG) of *celR* containing the HTH and AAA^+^ domain was amplified from Bt HD73 genomic DNA using PCR with the primer pair CelRHA-F/CelRHA-R. The PCR product was digested and ligated into *Eco*RI/*Sal*I digested pET21b. The recombinant plasmid pET-HA was transferred into *E. coli* BL21 (DE3), yielding the strain BL21 (pET-HA), which produced hexahistidine-tagged CelR-HA. The strain was grown to an exponential phase in the LB medium. Expression and purification of the HA-His protein were performed as previously described (Peng et al., 2014).

### 4.8 Gel mobility shift assay

PCR amplification of DNA fragments from HD73 genomic DNA was performed using specific primers labeled with a 6-FAM modification at the 5′-end: PcelA-F-FAM/PcelA-R-FAM. The PCR products were purified with a PCR cleanup kit (AXYGEN, Hangzhou, China). Purified CelR-HA and CcpA were incubated with the fluorescently-labeled DNA fragment (1.5 ng) in 10 μL of binding buffer containing 10 mM Tris–HCl, 0.5 mM EDTA, 0.5 mM dithiothreitol (DTT), 50 mM NaCl, and 4% (vol/vol) glycerol. After incubation for 20 min, the reaction mixture was submitted to electrophoresis at 4°C in TBE buffer (89 mM Tris base, 89 mM boric acid, and 1 mM EDTA [pH 8.0]) for 1 h at 120 V. The gel was photographed using Typhoon 9410 (GE Healthcare, Massachusetts, USA).

### 4.9 β-Galactosidase assays

The experiments were conducted in SSM medium, as described previously (Peng et al., 2014) and compared with LB when appropriate. *Bt* strains containing *lacZ* transcriptional fusions were grown in SSM and LB medium supplemented with 1 mM cellobiose at 30°C under shaking (220 rpm) until the end of the exponential phase (OD600 = 2.0–2.2, corresponding to T_0_), and then every hour for 8 h (T_1_ to T_8_). For the CCR part, Cellobiose (1 mM) was added to the medium at the mid-exponential phase (T_−1_) in SSM, and 7 mM glucose supplementation is specifically used. Two ml of the samples were collected at 1-h intervals after induction. Cells were harvested by centrifugation for 1 min at 12,000 × g, and the supernatant was discarded. β-Galactosidase activities were measured as described previously (Peng et al., 2014). The reported values represent the averages from at least three independent assays. The data were analyzed by *t*-test using the SPSS software (version 19.0). Error bars represent standard deviations.

### 4.10 Cellobiose utilization

*Bt* strains were cultured in LB medium at 30°C and 220 rpm until the end of the exponential phase (OD600 = 3.0). The cells were centrifuged to remove the supernatant and suspended in the same volume of M9 medium. The cells at the inoculation amount (1%) were transferred into the M9 medium, to which we added 1% cellobiose (M/V) as the sole carbon source at 30°C and 220 rpm. Next, OD600 was measured at 3-h intervals. Values are reported as the mean of at least three independent assays.

### 4.11 PTS activity

The PEP-dependent PTS activity assay was carried out as described by Wu et al. (2012). Briefly, *Bt* strains were cultured overnight in 50 mL of LB and SSM medium with or without 0.3 mM cellobiose at 30°C under shaking (220 rpm) until the mid-exponential phase. Cells were harvested by centrifugation, and the supernatant was discarded. Bacteria were washed twice with 0.1 M PBS buffer and resuspended in 5 mL of the same buffer. Each sample was combined with 250 μL of the toluene-acetone (1:9) mixture and vortexed vigorously for 5 min. The samples were then frozen and stored until further use. One milliliter of the sample was freeze-dried, and measured to evaluate the dry weight of the cells. Each sample (50 μL) was mixed with 100 μL of 1 mM PEP, 100 μL of 1 mM β-NADH, 10 U of rabbit muscle lactic acid dehydrogenase, and 100 μL of 0.1 M cellobiose, and incubated for 20 min at 30°C. The PTS activity was monitored by measuring the absorbance at 340 nm, and the reaction mixture without PEP served as a control. PTS activity was calculated as described by Wu et al. (2012). Values are reported as the mean of at least three independent assays.

## Data availability statement

The original contributions presented in the study are included in the article/Supplementary material, further inquiries can be directed to the corresponding author.

## Author contributions

QP was designed and planned the study. QP and LZ were compiled the draft of the manuscript. LZ was mainly performed the experimental work. HX was performed complementary experimental work. HC was constructed the *cel* operon mutants. FS and JZ were critically revised the manuscript for intellectual content. All authors read and approved the final version of the manuscript.

## Footnotes

^1^ https://dbtbs.hgc.jp/
