# Supplementary material for: Transcriptional regulation of cellobiose utilization by PRD-domain containing Sigma54-dependent transcriptional activator (CelR) and catabolite control protein A (CcpA) in Bacillus thuringiensis
Source: Front Microbiol. 2024 Jan 31;15:1160472. doi: 10.3389/fmicb.2024.1160472 (PMC10864463; doi:10.3389/fmicb.2024.1160472)
Supplement: Supplementary file 1 [file Presentation_1.pdf]

Transcriptional regulation of cellobiose utilization by PRD-domain containing Sigma54-dependent transcriptional activator (CelR) and catabolite control protein A (CcpA) in *Bacillus thuringiensis*

Liangwei Zhang[1#], Hong Xu[1#], Haijian Cheng[1], Fuping Song[1], Jie Zhang[1], Qi Peng[1*]

1 State Key Laboratory for Biology of Plant Diseases and Insect Pests, Institute of Plant Protection, Chinese Academy of Agricultural Sciences, Beijing, China

# These authors contributed equally to this work.

*** Correspondence:**
Qi Peng: qpeng@ippcaas.cn

Supplementary Figures and Tables

(A)

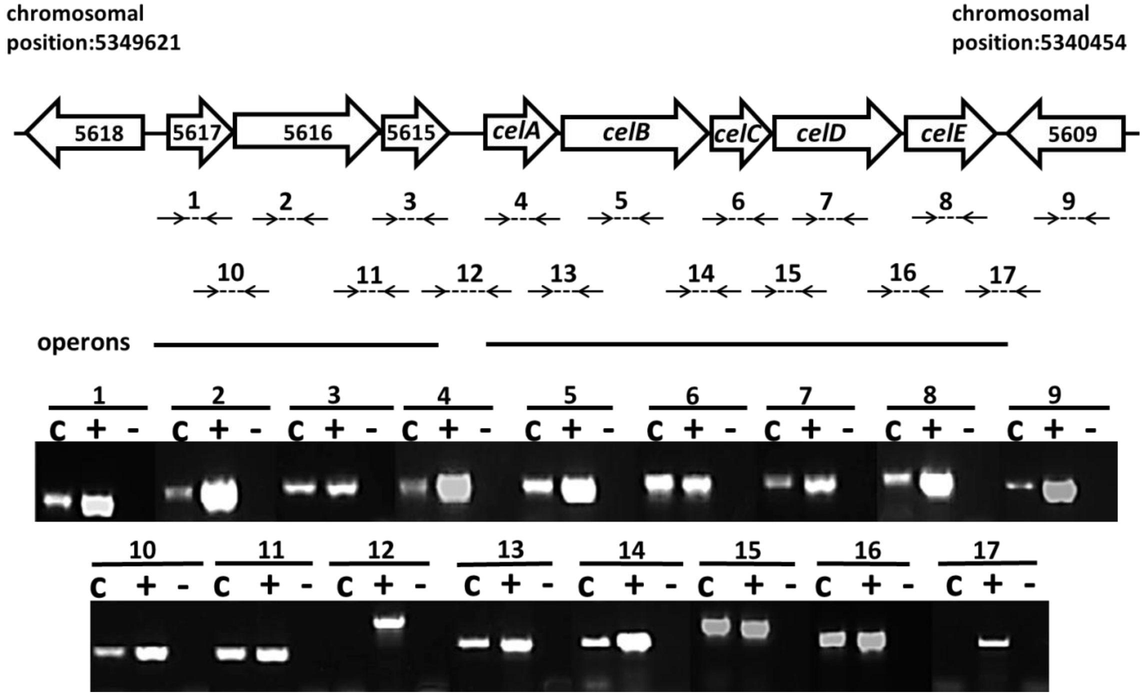

(B)

```
Adapter          AAGCAGTGGTATCAACGCAGAGT-ACATGGG
Clone1      TAGAGATTAAGCAGTGGTATCAACGCAGAGT-ACATGGGGTAAAAAAAAAATA
Clone2      TAGAGATTAAGCAGTGGTATCAACGCAGAGT-ACATGGGGTAAAAAAAAAATA
Clone3      TAGAGATTAAGCAGTGGTATCAACGCAGAGT-ACATGGGGTAAAAAAAAAATA
Clone4      TAGAGATTAAGCAGTGGTATCAACGCAGAGT-ACATGGGGTAAAAAAAAAATA
Clone5      TAGAGATTAAGCAGTGGTATCAACGCAGAGT-ACATGGGGTAAAAAAAAAATA
Clone6      TAGAGATTAAGCAGTGGTATCAACGCAGAGT-ACATGGGGTAAAAAAAAAATA
Clone7      TAGAGATTAAGCAGTGGTATCAACGCAGAGT-ACATGGGGTAAAAAAAAAATA
Clone8      TAGAGATTAAGCAGTGGTATCAACGCAGAGT-ACATGGGGTAAAAAAAAAATA
Clone9      TAGAGATTAAGCAGTGGTATCAACGCAGAGT-ACATGGGGTAAAAAAAAAATA
Clone10     TAGAGATTAAGCAGTGGTATCAACGCAGAGT-ACATGGGGTAAAAAAAAAATA
Clone11     TAGAGATTAAGCAGTGGTATCAACGCAGAGT-ACATGGGGTAAAAAAAAAATA
Clone12     TAGAGATTAAGCAGTGGTATCAACGCAGAGT-ACATGGGGTAAAAAAAAAATA
Clone13     TAGAGATTAAGCAGTGGTATCAACGCAGAGT-ACATGGGGTAAAAAAAAAATA
Clone14     TAGAGATTAAGCAGTGGTATCAACGCAGAGT-ACATGGGGTAAAAAAAAAATA
Clone15     TAGAGATTAAGCAGTGGTATCAACGCAGAGT-ACATGGGGTAAAAAAAAAATA
Clone16     TAGAGATTAAGCAGTGGTATCAACGCAGAGT-ACATGGGGTAAAAAAAAAATA
Clone17     TAGAGATTAAGCAGTGGTATCAACGCAGAGT-ACATGGGGTAAAAAAAAAATA
Clone18     TAGAGATTAAGCAGTGGTATCAACGCAGAGT-ACATGGGGTAAAAAAAAAATA
Clone19     TAGAGATTAAGCAGTGGTATCAACGCAGAGT-ACATGGGGTAAAAAAAAAATA
Clone20     TAGAGATTAAGCAGTGGTATCAACGCAGAGT-ACATGGGGTAAAAAAAAAATA

celA gene   ---TAAAAAGTTGGCACGCTAATTGCATTAATAAATAGCGTAAAAAAAAAATA---
                       -24        -12           +1
```

Supplementary Figure 1. RT-PCR and 5'RACE analysis of *cel* gene cluster. (A) RT-PCR analysis of *cel* gene cluster. RT-PCR analysis of the *cel* locus in Bt strain HD73. The RNA samples were prepared at T_7 of stationary phase (7 hours after the end of the exponential phase) in SSM. The white arrows represent ORFs. Dashed lines with small black arrows annotated with letters correspond to RT-PCR amplicons. The full lines below the ORFs indicate operons. The RT-PCR reactions labeled 'c' were performed with 500 ng RNA. The positive controls are labeled '+': PCR with 100 ng genomic DNA. The negative controls are labeled '-': RT-PCR with 500 ng RNA with heat-inactivated reverse transcriptase. **(B) 5'RACE analysis of *cel* gene cluster.** Underlining sequence of Clone1-20 represents the 3 terminal of adapter primer. The neighbored G residue is the *celA* start codon. The -24 /-12 sequences are marked in underlined.

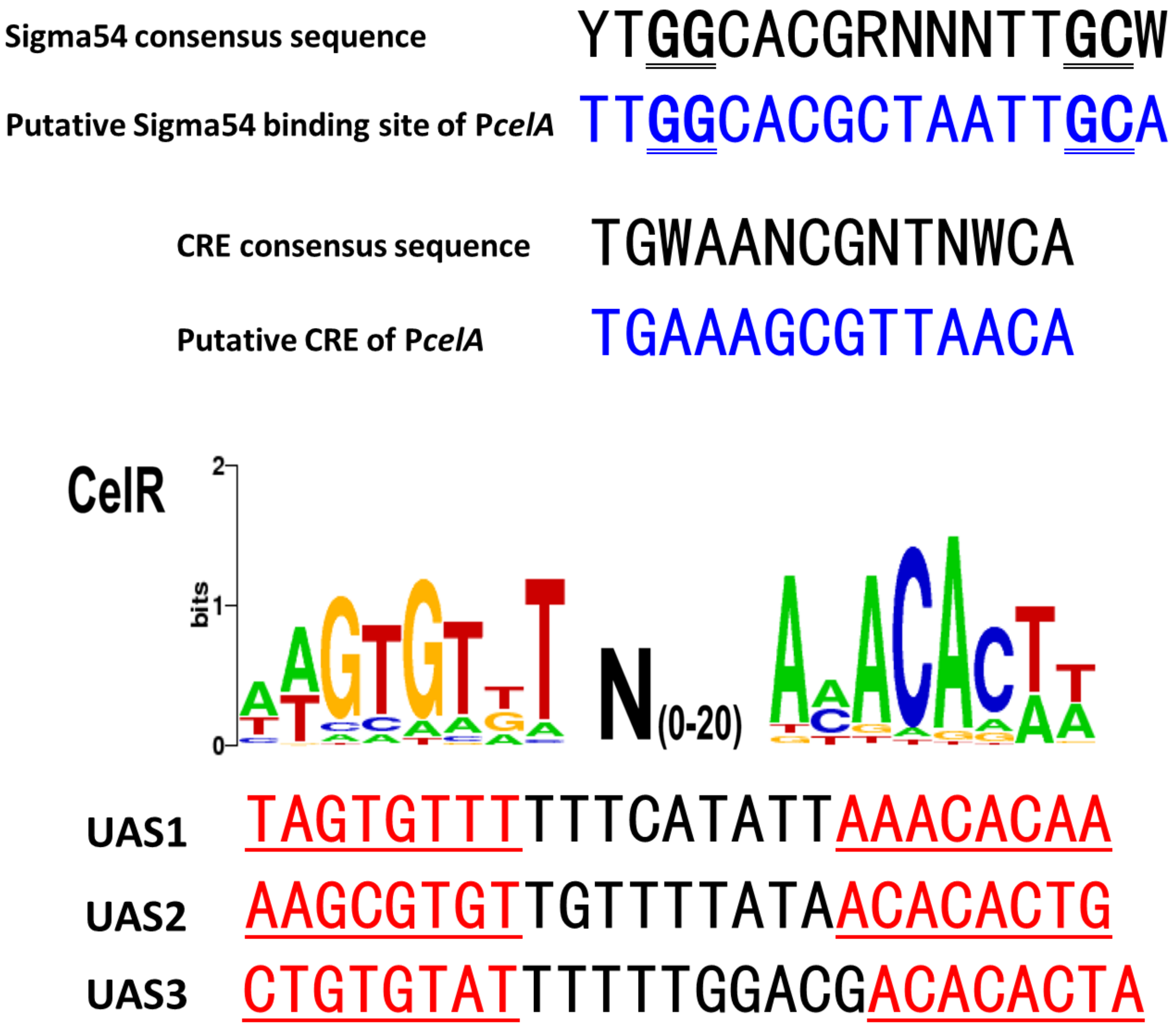

Supplementary Figure 2. The predicted DNA-binding sites for Sigma54, CcpA and CelR. N is nonconserved, Y is pyrimidines, R is purines, and W is A or T. Sigma54 consensus sequence is from reference 1. CRE consensus sequence is from reference 2. CelR consensus sequence is from reference 3.

1. Barrios H, Valderrama B, Morett E. Compilation and analysis of sigma(54)-dependent promoter sequences. Nucleic Acids Res. 1999 Nov 15;27(22):4305-13.
2. Weickert MJ, Chambliss GH. Site-directed mutagenesis of a catabolite repression operator sequence in *Bacillus subtilis*. Proc Natl Acad Sci U S A. 1990 Aug;87(16):6238-42.
3. Nie X, Yang B, Zhang L, Gu Y, Yang S, Jiang W, Yang C. PTS regulation domain-containing transcriptional activator CelR and sigma factor σ(54) control cellobiose utilization in *Clostridium acetobutylicum*. Mol Microbiol. 2016 Apr;100(2):289-302.

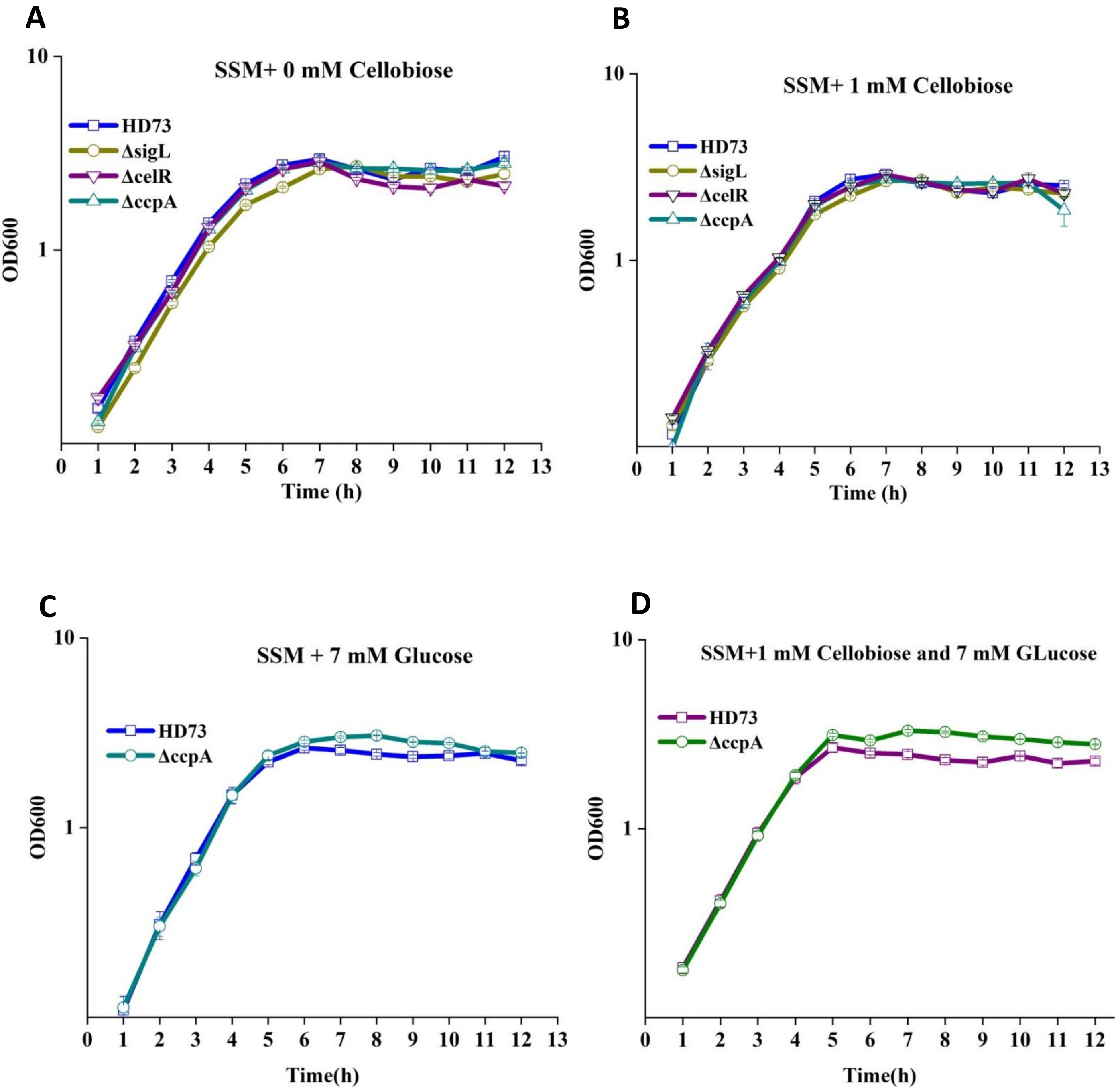

Supplementary Figure 3. Growth curve of Bt HD73 and *sigL*, *celR* and *ccpA* mutants.
The cells were cultured in the SSM medium with or without 1 mM cellobiose as the sole carbon at 30 ° C and 220 rpm. Next, OD600 was measured at 2 h intervals. The y axis represents OD600 in different strains. The x axis represents different time. (A) Strains were cultured in the SSM medium without cellobiose. (B) Strains were cultured in SSM medium with 1 mM cellobiose. (C) Strains were cultured in the SSM medium with 7 mM glucose. (D) Strains were cultured in the SSM medium with 7 mM glucose and 1 mM cellobiose.

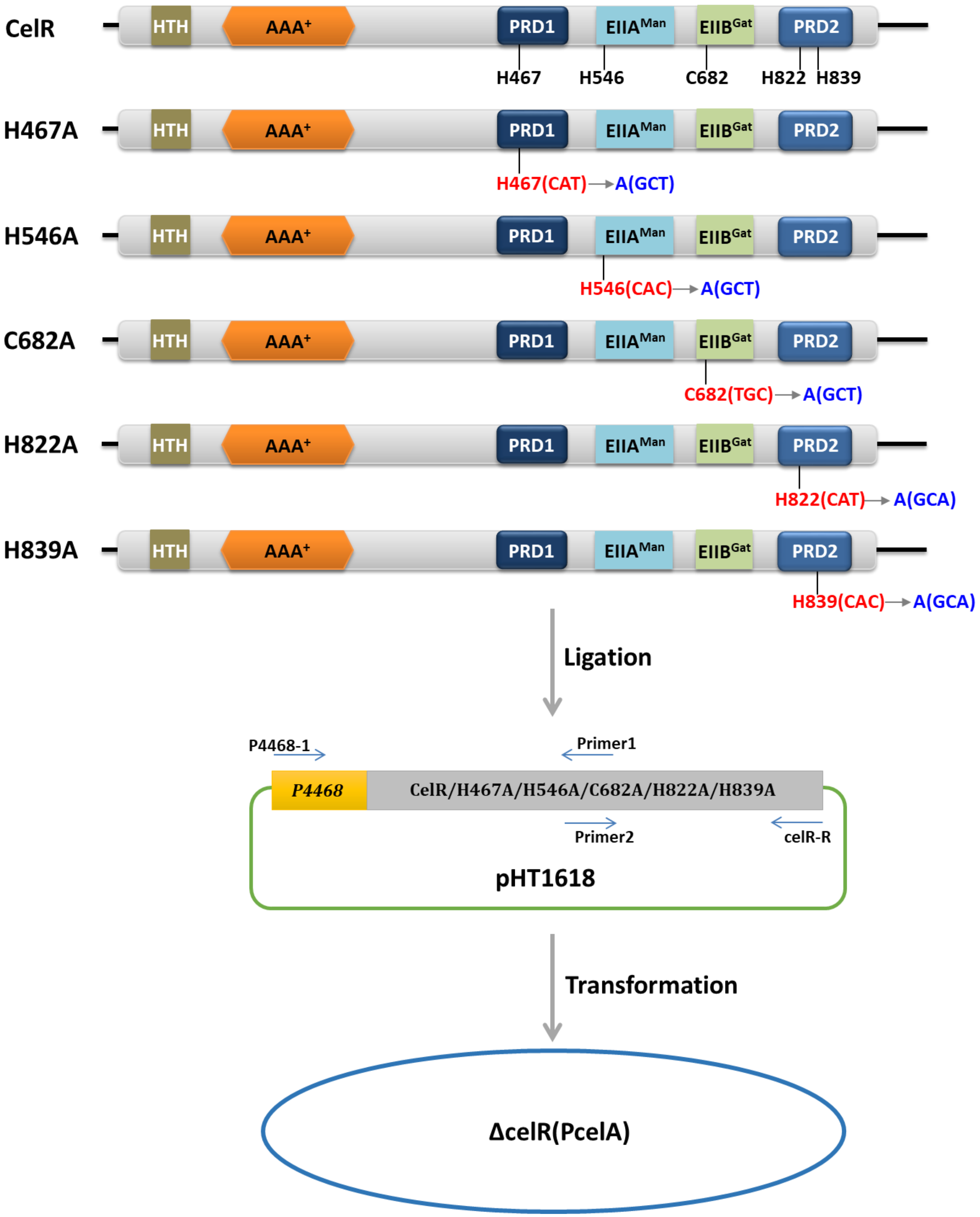

Supplementary Figure 4. The constructed schematic of PRD, EIIA and EIIB domains point-mutants.

High expression promoter P4468 was used to direct the expression of CelR. P4468 was amplified from HD73 genomic DNA using PCR with the primer pair P4468-1/P4468-2. *celR* fragment was amplified from HD73 genomic DNA using PCR with celR-F/celR-R as primers. The PCR products were purified and ligated into the plasmid pHT1618 using a seamless cloning kit. The resulting plasmid (pHT-CcelR) was introduced into the *Bt* mutant strain Δ*celR*(P*celA*), yielding the complemented strain C*celR*(P*celA*). This strain complements the *celR* mutant, allowing us to evaluate the expression of the *celA* promoter with a *lacZ* fusion.

The upstream fragment (2,152 bp) and the downstream fragment (1,317 bp) of the *celR* gene containing the mutated site of His-467 were amplified using PCR with the plasmid pHT-CcelR as the template and the primers P4468-1/H1U-R and H1D-F/celR-R, respectively. CelR fragments (3,208 bp and 246 bp) with a mutation at His-822 were amplified using PCR with the plasmid pHT-CcelR as the template and P4468-1/H2U-R and H2D-F/celR-R as the primer pairs. CelR fragments (3,242 bp and 173 bp) with a mutation at His-839 were amplified using PCR with the plasmid pHT-CcelR as the template and P4468-1/H839U-R and H839D-F/celR-R as the primer pairs. CelR fragments (2,386 bp and 1078 bp) with a mutation at His-546 were amplified using PCR with the plasmid pHT-CcelR as the template and P4468-1/H546U-R and H546D-F/celR-R as the primer pairs. CelR fragments (2,794 bp and 673 bp) with a mutation at Cys-682 on were amplified using PCR with the plasmid pHT-CcelR as the template and P4468-1/H682U-R and H682D-F/celR-R as the primer pairs. Next, the PCR products were purified and ligated into the plasmid pHT1618 using a seamless cloning kit. The resulting plasmids (pHT-*celR*-H467A, pHT-*celR*-H822A, pHT-*celR*-H839A, pHT-*celR*-H546A, and pHT-*celR*-C682A) were introduced into the *Bt* mutant strain Δ*celR*(P*celA*), yielding the strains H467A(P*celA*), H822A(P*celA*), H839A(P*celA*), H546A(P*celA*), and C682A(P*celA*), respectively. These plasmids complement the *celR* mutant strain without the His467, His822, His839, His546, and Cys682 residues, respectively, allowing us to evaluate the expression of the *celA* promoter using *lacZ* fusion constructs.

Strains	Gene-ID	Amino acid sequence of PRD1	Amino acid sequence of EIIA	Amino acid sequence of EIIB	Amino acid sequence of PRD2
Bacillus thuringiensis serovar kurstaki str. HD73	HD73_5607(CelR)	...LALSLHVQTTL...	...VLILAHGNGIA...	...VILTACLTGEG...	...MGVILHMSCMVDRLQKG.ESLLPHPDK...
Bacillus subtilis subsp. subtilis str. 168	BSU27080(LevR)	...YFLSMHIDAFL...	...IIVAAHGNSTA...	...ALVSICTTGSG...	...IKVIMHTAFAFERVIKQ..NPIAFSEE...
Clostridium acetobutylicum ATCC 824	CA_C0382(CelR)	...TALALHINTLI...	...IIIIAHGKSTA...	...AIVTACLTGEG...	...IGLVIHIAFAISRLKKG.SHSAEYPDK...
Bacillus anthracis str. 'Ames Ancestor	GBAA_5437(CelR)	...LALSLHVQTTL...	...VLILAHGNGIA...	...VILTACLTGEG...	...MGVILHMSCMVDRLQKG.ESLLPHPDK...
Bacillus cereus ATCC 14579	BC5205(CelR)	...LALSLHVQTTL...	...VLILAHGNGIA...	...VILTACLTGEG...	...MGVILHMSCMVDRLQKG.ESLLPHPDK...
Bacillus cereus strain BHU1	CPZ31_26040(CelR)	...LALSLHVQTTL...	...VLILAHGNGIA...	...VILTACLTGEG...	...MGVILHMSCMVDRLQKG.ESLLPHPDK...
Bacillus cereus strain A1	DA68_21925(CelR)	...LALSLHVQTTL...	...VLILAHGNGIA...	...VILTACLTGEG...	...MGVILHMSCMVDRLQKG.ESLLPHPDK...
Bacillus cereus strain A22	FHP23_025955(CelR)	...LALSLHVQTTL...	...VLILAHGNGIA...	...VILTACLTGEG...	...MGVILHMSCMVDRLQKG.ESLLPHPDK...
Bacillus cereus strain J7	L6482_RS26040(LevR)	...LALSLHVQTTL...	...VLILAHGNGIA...	...VILTACLTGEG...	...MGVILHMSCMVDRLQKG.ESLLPHPDK...
Bacillus cereus strain M3	BA202_RS26345(LevR)	...LALSLHVQTTL...	...VLILAHGNGIA...	...VILTACLTGEG...	...MGVILHMSCMVDRLQKG.ESLLPHPDK...
Bacillus thuringiensis str. Al Hakam	BALH_4697(CelR)	...LALSLHVQTTL...	...VLILAHGNGIA...	...VILTACLTGEG...	...MGVILHMSCMVDRLQKG.ESLLPHPDK...
Bacillus thuringiensis strain H3	G9298_26780(CelR)	...LALSLHVQTTL...	...VLILAHGNGIA...	...VILTACLTGEG...	...MGVILHMSCMVDRLQKG.ESKLPHPDK...
Bacillus thuringiensis strain ABTS-1857	K8Z20_RS26600(CelR)	...LALSLHVQTTL...	...VLILAHGNGIA...	...VILTACLTGEG...	...MGVILHMSCMVDRLQKG.ESLLPHPDK...
Bacillus thuringiensis str. YBT-1520	YBT1520_27750(LevR)	...LALSLHVQTTL...	...VLILAHGNGIA...	...VILTACLTGEG...	...MGVILHMSCMVDRLQKG.ESLLPHPDK...
Bacillus thuringiensis strain S38	NGM22_RS22100(LevR)	...LALSLHVQTTL...	...VLILAHGNGIA...	...VILTACLTGEG...	...MGVILHMSCMVDRLQKG.ESLLPHPDK...
Bacillus thuringiensis Bt407	BTB_RS26515(LevR)	...LALSLHVQTTL...	...VLILAHGNGIA...	...VILTACLTGEG...	...MGVILHMSCMVDRLQKG.ESLLPHPDK...
Bacillus thuringiensis strain ATCC 10792	CAB88_RS27030(LevR)	...LALSLHVQTTL...	...VLILAHGNGIA...	...VILTACLTGEG...	...MGVILHMSCMVDRLQKG.ESLLPHPDK...
Bacillus sp. SH8-8	DN405_25600(CelR)	...LALSLHVQTTL...	...VLILAHGNGIA...	...VILTACLTGEG...	...MGVILHMSCMVDRLQKG.ESLLPHPDK...
Bacillus sp. JAS24-2	DN407_25710(CelR)	...LALSLHVQTTL...	...VLILAHGNGIA...	...VILTACLTGEG...	...MGVILHMSCMVDRLQKG.ESLLPHPDK...
Bacillus sp. MYb78	CQ064_RS12600(LevR)	...LALSLHVQTTL...	...VLILAHGNGIA...	...VILTACLTGEG...	...MGVILHMSCMVDRLQKG.ESLLPHPDK...
Bacillus albus strain PFYN01	EJW27_01490(CelR)	...LALSLHVQTTL...	...VLILAHGNGIA...	...VILTACLTGEG...	...MGVILHMSCMVDRLQKG.ESLLPHPDK...
Bacillus mycoides strain ATCC 6462	BG05_845(CelR)	...LALSLHVQTTL...	...VLILAHGNGIA...	...VILTACLTGEG...	...MGVILHMSCMVDRLQKG.ESLLPHPDK...
Bacillus mycoides KBAB4	BcerKBAB4_4997(CelR)	...LALSLHISSFL...	...VLILAHGNGIA...	...VILTACLTGEG...	...MGVILHMSCMVDRLQKG.ESLLPHPDK...
Bacillus toyonensis strain BPN45/4	EXW33_11315(CelR)	...LALSLHVQTTL...	...VLILAHGNGIA...	...VILTACLTGEG...	...MGVILHMSCMVDRLQKG.ESKLPHPDK...
Bacillus toyonensis strain JAS411	EXW25_RS26030(LevR)	...LALSLHVQTTL...	...VLILAHGNGIA...	...VILTACLTGEG...	...MGVILHMSCMVDRLQKG.ESLLPHPDK...
Bacillus luti strain FJ	EUC40_RS01080(LevR)	...LALSLHVQTTL...	...VLILAHGNGIA...	...VILTACLTGEG...	...MGVILHMSCMVDRLQKG.ESLLPHPDK...
Bacillus fungorum strain 17-SMS-01 ZB100004	CO726_RS09340(LevR)	...LALSLHVQTTL...	...VLILAHGNGIA...	...VILTACLTGEG...	...MGVILHMSCMVDRLQKG.ESLLPHPDK...
Bacillus pacificus strain BC875	NJ102_RS06695(LevR)	...LALSLHVQTTL...	...VLILAHGNGIA...	...VILTACLTGEG...	...MGVILHMSCMVDRLQKG.ESLLPHPDK...
Bacillus albus strain PG1	ETJ66_RS18590(LevR)	...LALSLHVQTTL...	...VLILAHGNGIA...	...VILTACLTGEG...	...MGVILHMSCMVDRLQKG.ESLLPHPDK...
Bacillus anthracis str. Ames	BA_RS25640(LevR)	...LALSLHVQTTL...	...VLILAHGNGIA...	...VILTACLTGEG...	...MGVILHMSCMVDRLQKG.ESLLPHPDK...
Bacillus anthracis str. A0248	BAA_RS27030(LevR)	...LALSLHVQTTL...	...VLILAHGNGIA...	...VILTACLTGEG...	...MGVILHMSCMVDRLQKG.ESLLPHPDK...
Bacillus anthracis str. H9401	H9401_RS26475(LevR)	...LALSLHVQTTL...	...VLILAHGNGIA...	...VILTACLTGEG...	...MGVILHMSCMVDRLQKG.ESLLPHPDK...
Clostridium beijerinckii NCIMB 8052	Cbei_3875(LevR)	...YATSLHFSALF...	...IVVASHGASTA...	...VIITVCSTGKG....	...LRIMFHVACALERMVLN..NGLVYDD....
Clostridium beijerinckii NCIMB 8052	Cbei_4915(LevR)	...YAISLHFSALF...	...IVVASHGTSTA...	...AIITICSTGKG....	...LRIMFHVACALERMLLN..NGLVYDS....
Clostridium beijerinckii NCIMB 8052	Cbei_4686(CelR)	...YGLAVHISNSI...	...IIIIAHGISTA...	...AIVTVCTTGEG...	...IGITLHMACVIDRYKEG.KVTLEFEGK...
Clostridium beijerinckii NCIMB 8052	Cbei_4641(CelR)	...TAFALHINTLI...	...VILIAHGESTA...	...LIVTACLTGEG...	...VGVILHLSFVIGRLRNE.DNPAVYPQK...
Clostridium beijerinckii NCIMB 8052	Cbei_4539(CelR)	...FGLALHLQGSI...	...VLVMMHGTSTA...	...VIITICFTGEG...	...IGILIHITFLIDKLKHG.GQELKFKDL...
Clostridium beijerinckii NCIMB 8052	Cbei_0953(CelR)	...YGLSMHVASTV...	...IIVAMHGEYTA...	...VIITACITGQG...	...TGLILHIACVIERMLSG.EKIIYRKYR...
Clostridium saccharoperbutylacetonicum N1-4	Cspa_c53790(CelR)	...YGLAVHINTLI...	...VIVIAHGVSTA...	...AIVTVCTTEGE...	...IGITLHIAAMIDMSIFK.KTIIEFEGK...
Clostridium saccharoperbutylacetonicum N1-4	Cspa_c49170(CelR)	...YGLAVHINNSI...	...IIIIAHGVSTA...	...AIITICTTGEG...	...IGIALHMSCMIDRLLEG.KNIDDFQGK...
Clostridium saccharoperbutylacetonicum N1-4	Cspa_c17380(CelR)	...YGLAVHISNSI...	...VIIIAHGEGTA...	...AIITVCTTGEG...	...IGITLHMAVMIDRLCGD.QGVDAYNNM...
Clostridium saccharoperbutylacetonicum N1-4	Cspa_c53690(CelR)	...TAFALHINTLI...	...VILIAHGDSTA...	...LIITACTTGEG...	...VGIILHIAFVIGRNE.NNPAIYPERDN...
Clostridium saccharoperbutylacetonicum N1-4	Cspa_c55920(LevR)	...YGLAVHISNSI...	...IVVAAHGTSTA...	...VIVTICSTGKG....	...LRIIFHVACALERMLLN..NGLVYDS....
Clostridium botulinum A str. ATCC 3502	CBO3358(CelR)	...FGLALHLQQSV...	...ILVIMHGKATA...	...LIITACFTGHG...	...IGMILHISFMIDKIKNG.GLENNFENL...
Thermoanaerobacter sp. X514	Teth514_0261(CelR)	...YGLSMHVASSV...	...IIVAMHGEAAA...	...VIITACITGEG...	...IGLILHLACVVERILQG.KQLIHMKDT...
Thermoanaerobacter sp. X514	Teth514_0131(LevR)	...LTHFINYLLSE...	...SIIIAHGYSTA...	...AIIATCITGIG....	...ISLYIHVSVMIERLVMK..EPIVFYS....
Thermoanaerobacter tengcongensis MB4	TTE0331(CelR)	...LTHFIHYLLSE...	...IIVAMHGEAAA...	...VIITACITGEG....	...IGLILHIACVIERIIEG.KELTHVKN....
Listeria welshimeri serovar 6b str. SLCC5334	lwe0744(CelR)	...FAFSLHLQSTI...	...VLVATHGNNTA...	...AIVTICATGEG...	...IGMILHLAFLIERVKLG.AVDREFPDL...
Listeria innocua Clip11262	lin1832(CelR)	...FAFSLHLQSTI...	...VLVATHGNNTA...	...AVIFTCFTGEG...	...IGMILHLAFLIERVKLG.AVDREFPDL...
Listeria monocytogenes EGD-e	lmo1721(CelR)	...FAFSLHFSALF...	...VIVMMHGRSTA...	...AVIFTCFTGEG...	...IGMILHLAFLIERVKLG.AVDREFPDL...
Enterococcus faecalis V583	EF1010(CelR)	...FAFALHLCSTI...	...VFVLMHGSSTA...	...AVIVICFTGEG...	...AGIMIHLAFLVDALIKG.EETRNFPNL...
Enterococcus faecalis V583	EF0018(ManR)	...YAMSLHISSFL...	...IVVAAHGNSTA...	...AIIAICASGEG....	...INLIMHTSGMLERILRN..DTLTVSE....
Enterococcus faecium DO ctg654	EfaeDRAFT 2264(GfrR)	...LSYYLYQRS.....	...AMIAAHGYATA...	...ALITTCFTGIG....	...LALYVHVSCLIERLIRQ..MPIETYQ....
Enterococcus faecalis V583	EF2981(LevR)	...NILIVHLYSAV...	...VIVVAHGAATA...	...VLIISCLTGMG...	...LRFIIHCACMLERQLLN---.PEYDPTA...
Pediococcus pentosaceus ATCC 25745	PEPE 1514(ManR)	...YAMSLHISSFI...	...IVVAAHGSSTA...	...AIVAVCSTGEG....	...INLIMHIAGAVERMLTN..NPMETAP....
Pediococcus pentosaceus SL4	T256_01095(GfrR)	...LSYYEYNRERS...	...CIILSHGYSTA...	...LIITCCFTGIG....	...MSLYVHISCLIERLIRN..EPITTYD....
Alkaliphilus metalliredigens QYMF	Amet_4197(CelR)	...YGLALHINCFI...	...ILVIMHGNTTA...	...IIIIACFTGEA...	...IGIILHISFLLDKLKSG.GSQTDFNEL...
Bacillus licheniformis ATCC 14580	BL03125(LevR)	...YFLSMHIAAFL...	...IVVAAHGNSTA...	...AIVSICTTGGG...	...IKVIMHTAFAFERVIKQ..NPIAFPEE...
Bacillus clausii KSM-K16	ABC4075(GfrR)	...FTHFIFYSSNQ...	...CLVVAHGFSTA...	...AIITTCVTGIG....	...ISMYVHLSCLIERLVTQ..RPIDSYP....
Brevibacillus brevis NBRC 100599	BBR47_56680(CelR)	...FGLALHIQTSK...	...VLVITHGSGGA...	...TIVVACLSGEG...	...IGAYLHICCLVDRLKAG.TQSVAYIQK...
Geobacillus thermodenitrificans NG80-2	GTNG_1199(CelR)	...YALALHIQTAI...	...VVVVMHGNGVA...	...AIVTACFTGKG...	...VGLVLHLCCLLDRLLSGEKTSSGEKTS...
Lactiplantibacillus plantarum JDM1	JDM1_0527(ManR)	...YAVSLHVSSFI...	...VLVAAHGMSTA...	...AIIAICATGVG....	...IDLIMHLAGALERTAIK..DEISAPT....
Lacticaseibacillus paracasei	LCABL_03890(EsuR)	...FASFIYLKQEV...	...AIILAHGYSTA...	...MIITTCHTGIG....	...VTLYVHVSAMIERLIRN..EPPLVYK....

Supplementary Figure 5. Sequence alignment of conserved histidyl or cysteine residues of CelR in Bt HD73 compared to PRD containing proteins in other bacteria. The conserved histidyl or cysteine residues of all the strains are marked in pink, and the other histidyl residues on PRD2 of Bc group are marked in green.

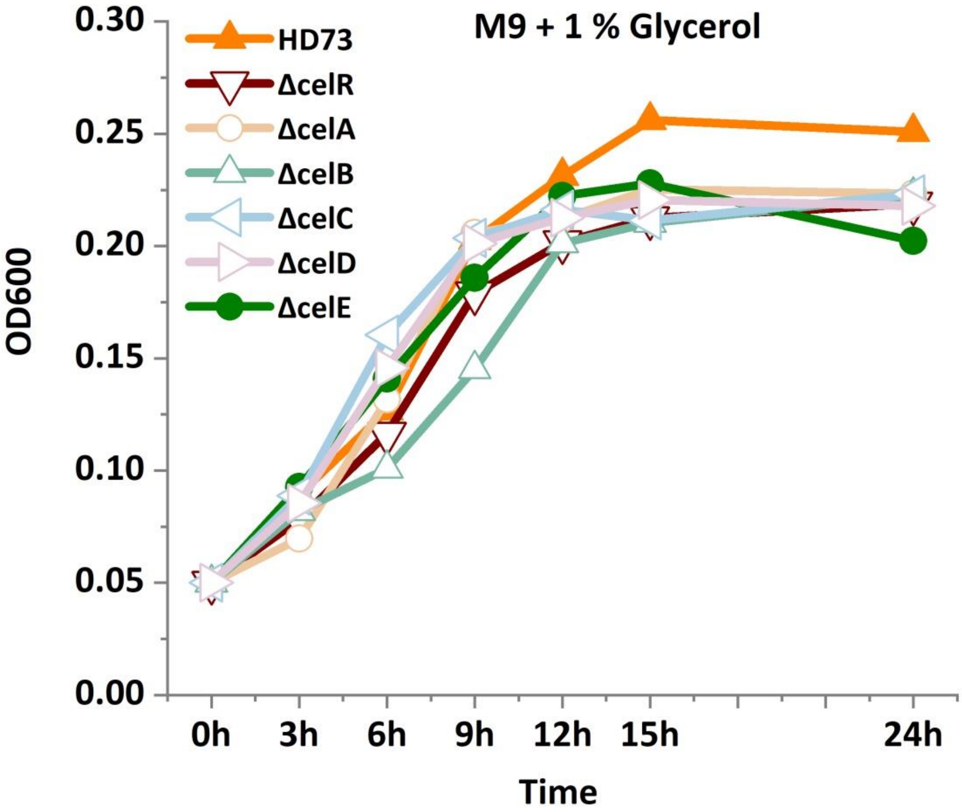

Supplementary Figure 6. Growth curve of Bt HD73 and *cel* mutants.
The cells were cultured in the M9 medium which was added 1% glycerol (M/V) as the sole carbon at 30 ° C and 220 rpm. Next, OD600 was measured at 3 h intervals. The *y* axis represents OD600 in different strains. The *x* axis represents different time.

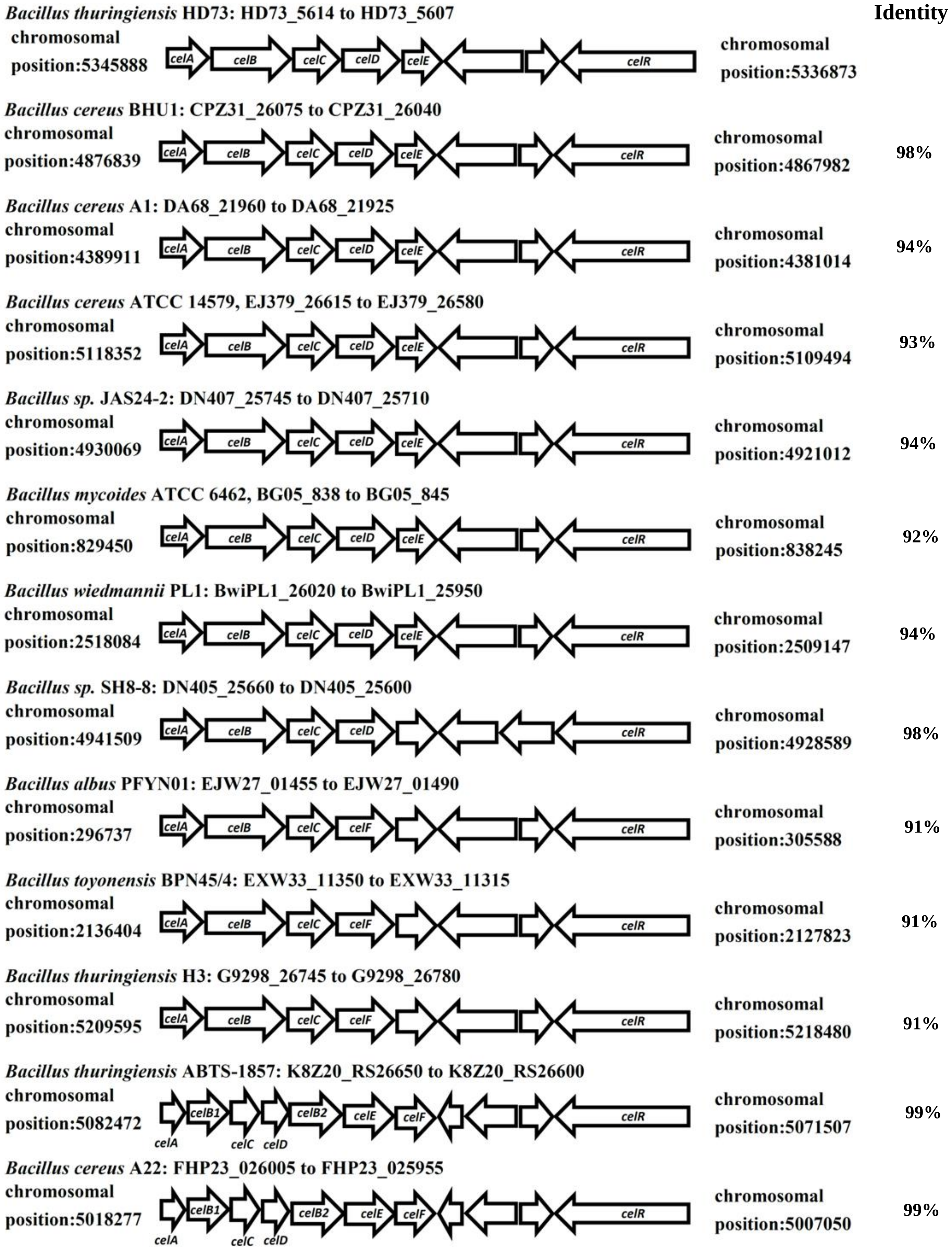

Supplementary Figure 7. The structure of the *cel* locus in different *Bacillus* species. Arrows represent open reading frame. *celA* encodes cellobiose-specific IIB component. *celB* encodes cellobiose-specific IIC component. *celC* encodes cellobiose-specific IIA component. *celD* and *celF* encode 6-phospho-beta-glucosidase. *celE* encodes YdjC-like protein.

Supplementary Table 1. Strains and plasmids used in this study

Strain or plasmid	Relevant genotype and characteristics	Reference or source
**Strains**		
HD73	*B. thuringiensis* subsp. *kurstaki* carrying the *cry1Ac* gene	This lab
Δ*sigL*	*B. thuringiensis* HD73 *sigL* gene mutant	(Zhu et al., 2010)
HD(pMADΔ*celR*)	Bt HD73 carrying pMADΔ*celR* plasmid,Kan^R, Em^R	This study
Δ*celR*	*B. thuringiensis* HD73 *celR* gene mutant	This study
HD(pMADΔ*celA*)	Bt HD73 carrying pMADΔ*celA* plasmid, Em^R	This study
HD(pMADΔ*celB*)	Bt HD73 carrying pMADΔ*celB* plasmid, Em^R	This study
HD(pMADΔ*celC*)	Bt HD73 carrying pMADΔ*celC* plasmid, Em^R	This study
HD(pMADΔ*celD*)	Bt HD73 carrying pMADΔ*celD* plasmid, Em^R	This study
HD(pMADΔ*celE*)	Bt HD73 carrying pMADΔ*celE* plasmid, Em^R	This study
Δ*celA*	*B. thuringiensis* HD73 *celA* gene mutant	This study
Δ*celB*	*B. thuringiensis* HD73 *celB* gene mutant	This study
Δ*celC*	*B. thuringiensis* HD73 *celC* gene mutant	This study
Δ*celD*	*B. thuringiensis* HD73 *celD* gene mutant	This study
Δ*celE*	*B. thuringiensis* HD73 *celE* gene mutant	This study
Δ*ccpA*	*B. thuringiensis* HD73 *ccpA* gene mutant	(Peng et al., 2020)
HD(P*celA*)	Bt HD73 carrying p18Z-P*celA* plasmid, Em^R	This study
Δ*sigL*(P*celA*)	Δ*sigL* carrying p18Z-P*celA* plasmid, Em^R	This study
Δ*celR*(P*celA*)	Δ*celR* carrying p18Z-P*celA* plasmid, Em^R	This study
Δ*ccpA*(P*celA*)	Δ*ccpA* carrying p18Z-P*celA* plasmid, Em^R	This study
CcelR(P*celA*)	Δ*celR* carrying p18Z-P*celA* and pHT-CcelR plasmid, Em^R and Tet^R	This study
H467A(P*celA*)	Δ*celR* carrying p18Z-P*celA* and pHT-*celR*-H467A plasmid, Em^R and Tet^R	This study
H546A(P*celA*)	Δ*celR* carrying p18Z-P*celA* and pHT-*celR*-H546A plasmid,	This study

	Em^R and Tet^R	
H822A(P*celA*)	Δ*celR* carrying p18Z-P*celA* and pHT-*celR*-H822A plasmid, Em^R and Tet^R	This study
H839A(P*celA*)	Δ*celR* carrying p18Z-P*celA* and pHT-*celR*-H839A plasmid, Em^R and Tet^R	This study
C682A(P*celA*)	Δ*celR* carrying p18Z-P*celA* and pHT-*celR*-C682A plasmid, Em^R and Tet^R	This study
HD(ΔUAS1)	*B. thuringiensis* HD73 contain p18Z-UAS1, Em^R	This study
HD(ΔUAS2)	*B. thuringiensis* HD73 contain p18Z-UAS2, Em^R	This study
HD(ΔUAS3)	*B. thuringiensis* HD73 contain p18Z-UAS3, Em^R	This study
HD(ΔUAS123)	*B. thuringiensis* HD73 contain p18Z-UAS123, Em^R	This study
E. coli TG1	Δ(*lac-proAB*) *supE thi hsd*-5 (*F' traD36 proA*$^+$ *proB*$^+$ *lacI*^{q} *lacZ*ΔM15), general purpose cloning host	Laboratory collection
E. coli ET12567	*F*$^-$ *dam-13*::Tn*9 dcm-6 hsdM hsdR recF143 zjj-202*::Tn*10 galK2 galT22 ara14 pacY1 xyl-5 leuB6 thi-1*, for generation of unmethylated DNA	Laboratory collection
E.coli BL21(DE3)	*E.coli B, F*$^-$*, dcm, ompT, hsdS(rB-mB-), gal, λ(DE3)*	Laboratory collection
BL21 (pET-HA)	BL21(DE3) with pET-HA plasmid	This study
BL21 (pET-ccpA)	BL21(DE3) with pET-ccpA plasmid	(Peng et al., 2020)
**Plasmids**		
pMAD	shuttle vector, thermosensitive origin of replication, Ap^R, Em^R	(Arnaud et al., 2004)
pMADΔ*celA*	pMAD with *celA* deletion fragment, Ap^R, Em^R	This study
pMADΔ*celB*	pMAD with *celB* deletion fragment, Ap^R, Em^R	This study
pMADΔ*celC*	pMAD with *celC* deletion fragment, Ap^R, Em^R	This study
pMADΔ*celD*	pMAD with *celD* deletion fragment, Ap^R, Em^R	This study
pMADΔ*celE*	pMAD with *celE* deletion fragment, Ap^R, Em^R	This study
pMADΔ*celR*	pMAD with *celR* deletion fragment, Ap^R, Em^R	This study

pHT304-18Z	Promoterless *lacZ* Vector, Em^R, Ap^R	(Agaisse and Lereclus, 1994)
P18Z-P*celA*	pHT304-18Z carrying *celA* promoter, Ap^R, Em^R	This study
P18Z-UAS1	pHT304-18Z carrying *celA* promoter mutated in UAS1, Ap^R, Em^R	This study
p18Z-UAS2	pHT304-18Z carrying *celA* promoter mutated in UAS2, Ap^R, Em^R	This study
p18Z-UAS3	pHT304-18Z carrying *celA* promoter mutated in UAS3, Ap^R, Em^R	This study
p18Z-UAS123	pHT304-18Z carrying *celA* promoter mutated in UAS1, UAS2 and UAS3, Ap^R, Em^R	This study
pHT1618	*E.coli-B.thuringiensis* shuttle vector, Ap^R, Tet^R	(Lereclus and Arantes, 1992)
pHT-CcelR	pHT1618 containing P4468 promoter and *celR* gene, Ap^R, Tet^R	This study
pHT-*celR*-H467A	pHT1618 containing P4468 promoter and *celR* where histidine-467 were replaced by alanine residue, Ap^R, Tet^R	This study
pHT-*celR*-H546A	pHT1618 containing P4468 promoter and *celR* where histidine-546 were replaced by alanine residue, Ap^R, Tet^R	This study
pHT-*celR*-H822A	pHT1618 containing P4468 promoter and *celR* where histidine-822 were replaced by alanine residue, Ap^R, Tet^R	This study
pHT-*celR*-H839A	pHT1618 containing P4468 promoter and *celR* where histidine-839 were replaced by alanine residue, Ap^R, Tet^R	This study
pHT-*celR*-C682A	pHT1618 containing P4468 promoter and *celR* where cysteine-682 were replaced by alanine residue, Ap^R, Tet^R	This study
pET21b	Expression vector, 5.4 kb, Ap^R	Laboratory collection

pET-HA	pET21b containing HTH and AAA+ domain of *celR*, Ap^R	This study

Supplementary Table 2: Primers used in this study

Oligonucleotides	Sequence (5' - 3') [a]	Used for
RT1-F	AACAATCTGGGCAGTATCAGGTT	RT-PCR analysis
RT1-R	TCATGGTTTCATTAACAAAGC	
RT2-F	AGATGGAATTATTTTAACGATGC	
RT2-R	CACAAGCGACAATGCCCCG	
RT3-F	CGCGAGAAGTTGTTCAATGG	
RT3-R	TCATTCATCCACCGACATCTTCCTAT	
RT4-F	TGGAAGCGGCTGCAAAAG	
RT4-R	TTATTTTTTAAGTGTTAATGCGTGA	
RT5-F	TAGAATTGGATCGCAGCGC	
RT5-R	GCCGTAAGATTTCGCTAAATTGTAA	
RT6-F	AAGCTTTGCGATGGAGGC	
RT6-R	TTATTCCCCTTTCGCTTCAA	
RT7-F	TCCTGGTGGTTTATTTAAAGCG	
RT7-R	CTTACGCCATCTAAGTATACATCTA	
RT8-F	TACAAATTACGGCATTATTGATG	
RT8-R	AACTTCTTCTGGATTTATATTCCCT	
RT9-F	CGGCGTTAACACCAAGTAGTG	
RT9-R	GACAGTTTCTATCGCTCTGGCG	
RT10-F	CGCAAGTACGTTATTTATTACCGA	
RT10-R	GGCAGTGCACTAATAATGAGGAA	
RT11-F	AGTGGATATTTAGGATCGGGC	
RT11-R	CGCCTCCTTTAATTCCTCTAGTG	
RT12-F	CAATTACGGTGAAAGAATTAGC	
RT12-R	AGCTTTTGCAGCCGCTTC	
RT13-F	CATGCTCTCTTCAATGAAAACG	

RT13-R	TTGGTAAGTTATTTATAAGTACTGC	
RT14-F	TATGTACAGGCTGCAAAAGCTG	
RT14-R	CTTCGTGCGTTACCACTATTTA	
RT15-F	GCGCATCATTTCCAAACAGA	
RT15-R	TGCCTCACGGCGATCAAG	
RT16-F	GTACCATCAGATAAAGTTGCAAAAC	
RT16-R	TACTCTTTCGCTAAGCGTACCG	
RT17-F	CCAGCTTATATTGATCCAGAGCTTG	
RT17-R	CAGTTGTTGGTATTTTAATTGCCGC	
celA-a	CGGGAGCTCGAATTCCGCTAGCATTCTTCATTT	Construction of *celA* mutant
celA-b	GCTTCCCCCCTAATTACATTTGAAAATCCCCC	
celA-c	GGGGGATTTTCAAATGTAATTAGGGGGGAAGC	
celA-d	GGCGATATCGGATCCCCTTGTAATGGTAATTGA	
celB-a	CGGGAGCTCGAATTCGTCGGTGGATGAATGAACC	Construction of *celB* mutant
celB-b	TTTTTAAAGTGATACCTTTTGCATTTCGCT	
celB-c	AGCGAAATGCAAAAGGTATCAATTTAAAAA	
celB-d	GGCGATATCGGATCCGCCTCACGGCGATCAAGCG	
celC-a	CGGGAGCTCGAATTCCGGGTGCAACGTTAGCAT	Construction of *celC* mutant
celC-b	CATGCTTTATTCCCCA GTCATCATA ATTAC	
celC-c	GTAATTATGATGACTGGGGAATAAAGCATG	
celC-d	GGCGATATCGGATCCCACACGGTCCATTACACT	
celD-a	CGGGAGCTCGAATTCTACAGGCTGCAAAAGCTG	Construction of *celD* mutant
celD-b	CCGCTTTTACTTCTCTCCAGTCATGCTTTA	
celD-c	TAAAGCATGACTGGAGAGAAGTAAAAGCGG	
celD-d	GGCGATATCGGATCCGTCACACCATCCGCATAA	
celE-a	CGGGAGCTCGAATTCGGGAGCCAGACTTATTCG	Construction of *celE* mutant
celE-b	CTATTAAAACTTCACCTTAATCATCATAAT	
celE-c	ATTATGATGATTAAGGTGAAGTTTTAATAG	
celE-d	GGCGATATCGGATCCCCTCGTCTTATTCTTTGG	
celRA-F	CGGGATCCCCTTTACACTGGTGACCCTTC	Construction of

celRA-R	CACCTCAAATGGTTCGCTGTAGCGAGTTCTGATGCGGTT	*celR* mutant
celRB-F	GAGCGCCTACGAGGAATTTCGTTGCAACCAATTGAAAAC	
celRB-R	RCGGAATTCGAATGTAGAGGATTTTGAGGGA	
celR-kmF	AACCGCATCAGAACTCGCTACAGCGAACCATTTGAGGTG	
celR-kmR	GTTTTCAATTGGTTGCAACGAAATTCCTCGTAGGCGCTC	
PcelA-F	GCCAGTGCCAAGCTTACCTTGCTTATGCAAGGTTTTT	Construction of *celA* promoter with *lacZ* gene fusion
PcelA-R	TGGGGATCCTCTAGATTGAAAATCCCCCTTGTGTAG	
P4468-1	AGTGCCAAGCTTGCATGCCTGCAGGGGAAGTTTAGGGAGGATTT	Complementation of the *celR* mutant
P4468-2	AATGAGATCTATTCGTTTCATATTTTGTAATCAACCCTTTC	
celR-F	GAAAGGGTTGATTACAAAATATGAAACGAATAGATCTCATT	
celR-R	CGGGGATCCTCTAGAGTCGACTTAATTTTTCACAGGCTGA	
H1U-R	AGAGTTGTCTGTACAGCTAAACTTAAAGCG	Construction of point mutations in $EIIA^{Man}$ and $EIIB^{Gat}$ domains and PRD
H1D-F	CGCTTTAAGTTTAGCTGTACAGACAACTCT	
H2U-R	ACCATACAGCTCATAGCCAGAATAACACCC	
H2D-F	GGGTGTTATTCTGGCTATGAGCTGTATGGT	
H839U-R	CTTTTCTCTTTATCAGGAGCAGGAAGAAGGC	
H839D-F	GCCTTCTTCCTGCTCCTGATAAAGAGAAAAG	
H546U-R	AGTTGCGATGCCATTACCAGCTGCTAATATTAATAC	
H546D-F	GTATTAATATTAGCAGCTGGTAATGGCATCGCAACT	
C682U-R	CCTTCACCAGTTAAAGCAGCTGTTAAAATAACAG	
C682D-F	CTGTTATTTTAACAGCTGCTTTAACTGGTGAAGG	
PcelA-F-FAM	GCCAGTGCCAAGCTTACCTTGCTTATGCAAGGTTTTT	Gel mobility shift assay
PcelA-R-FAM	TGGGGATCCTCTAGATTGAAAATCCCCCTTGTGTAG	
CelRHA-F	GGGTCGGGATCCGAATTCATGAAACGAATAGATCTCA	Expression and purification of the $HTH\text{-}AAA^{+}$ domain of CelR
CelRHA-R	GGCCGCAAGCTTGTCGACTGGCAGCGTAACAGTCATTGG	
UPM	TAGAGATTAAGCAGTGGTATCAACGCAGAGT	5′-RACE analysis

GSP	CTGCTTCTCCATTCATCATGCC	

[a]Restriction enzyme sites are underscored.

References

Agaisse, H., and Lereclus, D. (1994). Structural and functional analysis of the promoter region involved in full expression of the *cryIIIA* toxin gene of *Bacillus thuringiensis*. *Mol Microbiol* 13(1)**,** 97-107. doi: 10.1111/j.1365-2958.1994.tb00405.x.

Arnaud, M., Chastanet, A., and Debarbouille, M. (2004). New vector for efficient allelic replacement in naturally nontransformable, low-GC-content, gram-positive bacteria. *Appl Environ Microbiol* 70(11)**,** 6887-6891. doi: 10.1128/AEM.70.11.6887-6891.2004.

Lereclus, D., and Arantes, O. (1992). *spbA* locus ensures the segregational stability of pTH1030, a novel type of gram-positive replicon. *Mol Microbiol* 6(1)**,** 35-46. doi: 10.1111/j.1365-2958.1992.tb00835.x.

Peng, Q., Zhao, X., Wen, J., Huang, M., Zhang, J., and Song, F. (2020). Transcription in the acetoin catabolic pathway is regulated by AcoR and CcpA in *Bacillus thuringiensis*. *Microbiol Res* 235**,** 126438. doi: 10.1016/j.micres.2020.126438.

Zhu, L., Peng, Q., Song, F., Jiang, Y., Sun, C., Zhang, J., et al. (2010). Structure and regulation of the *gab* gene cluster, involved in the gamma-aminobutyric acid shunt, are controlled by a sigma54 factor in *Bacillus thuringiensis*. *J Bacteriol* 192(1)**,** 346-355. doi: 10.1128/JB.01038-09.
